# Functional Outcomes of Prosthetic Rehabilitation in Older Adults With Tooth Loss: A Systematic Review and Meta‐Analysis

**DOI:** 10.1111/ger.70078

**Published:** 2026-05-17

**Authors:** Pedro Molinero‐Mourelle, Azize Bell, Najla Chebib, Manabu Kanazawa, Frauke Müller, Annika Neri, Takahiro Ono, Masako Shiramizu, Sayaka Tada

**Affiliations:** ^1^ Department of Conservative Dentistry and Prosthodontics, Faculty of Dentistry Complutense University of Madrid Madrid Spain; ^2^ Department of Reconstructive Dentistry and Gerodontology School of Dental Medicine, University of Bern Bern Switzerland; ^3^ Complutense University of Madrid, Ramon y Cajal Research Institute (IRYCIS) Madrid Spain; ^4^ Restorative Dental Sciences, Faculty of Dentistry, the University of Hong Kong Hong Kong China; ^5^ Department of Prosthetic Dentistry and Material Science University Medical Center of the Johannes Gutenberg University Mainz Mainz Germany; ^6^ Division of Gerodontology and Removable Prosthodontics University Clinics of Dental Medicine, University of Geneva Geneva Switzerland; ^7^ Department of Gerodontology and Oral Rehabilitation Institute of Science Tokyo Tokyo Japan; ^8^ Centre of Excellence in Precision Medicine and Digital Health, Department of Physiology, Faculty of Dentistry Chulalongkorn University Bangkok Thailand; ^9^ Department of Geriatric Dentistry Osaka Dental University Osaka Japan; ^10^ Department of Dental Hygiene Kyoto Koka University Junior College Kyoto Japan; ^11^ Faculty of Dentistry, National University of Singapore Singapore

**Keywords:** bite force, chewing, dental prosthesis, masticatory performance, OHRQoL, patient‐reported outcomes, tooth loss

## Abstract

**Objectives:**

To assess the effect of prosthodontic treatment and management on oral function and oral‐health‐related patient reported outcome measures (PROMs) in older adults with tooth loss.

**Methods:**

A systematic search of four databases identified clinical studies (≥ 10 participants) reporting functional or PROM outcomes in partially or fully edentulous adults ≥ 65 years rehabilitated with dental prostheses for ≥ 6 months. Pooled standardized mean differences in pre–post changes were estimated by prosthesis type and follow‐up using random‐effects models (REML), with heterogeneity assessed by Cochran's *Q* and *I* ^2^ (*p* < 0.05).

**Results:**

From an initial 7654 studies, 24 were included in this review, covering 1711 individuals. Fourteen studies assessed masticatory performance (MP), masticatory ability (MA), and maximum bite force (MBF). Complete dentures (CDs) showed modest functional improvement, while implant overdentures (IODs) consistently produced higher MP and MBF with significant long‐term improvements. Eighteen studies evaluated OHRQoL using OHIP variants, OIDP, and modified GOHAI‐11. IODs and fixed full‐arch prostheses showed the greatest improvements, while CDs and removable partial dentures were associated with smaller but positive changes. Subjective MA improved over 6–12 months post insertion in regardless groups, with high heterogeneity. OHRQoL showed short‐term benefits of implant‐supported prostheses, particularly for OHIP‐20, while OHIP‐14 results were inconsistent over time, indicating more reliable functional and patient‐reported outcomes with dental implants.

**Conclusions:**

Tooth loss leads to functional and psycho‐social limitations, and although restorative measures can alleviate these shortcomings, they require ongoing maintenance, highlighting the complexity of care in this vulnerable older population.

**Trial Registration:**

PROSPERO International prospective register of systematic reviews (CRD420251077683)

## 1 Introduction

Tooth loss remains a prevalent condition among older adults worldwide and is closely associated with impaired oral function, altered nutritional intake, and diminished general and oral health‐related quality of life (OHRQoL) [1, 2, 3, 4]. Edentulism is associated with an increased prevalence of chronic gastric inflammation, non‐insulin dependent diabetes, several cardiovascular conditions, and chronic kidney diseases [5]. In terms of well‐being, persons who have lost teeth have described feelings like “losing a part of your body”, “feeling like a failure”, “feeling dirty” or even “being embarrassed” [6]. Tooth loss can affect social life in many ways, such as less participation in social activities, fewer meetings with friends, less frequent visits to restaurants, or reduced physical activity [5, 7].

Although the populations in developed countries are nowadays retaining their natural dentition until late in life, tooth loss is still very prevalent, with an estimated 267 million edentulous persons worldwide [8]. In Switzerland, as an example of a western world developed country, in the populations' age strata of 85 years and above, on average 19 teeth are missing and 59.7% of persons are wearing conventional removable dental prostheses (RDP) [9]. The cumulative nature of tooth loss, combined with age‐related physiological changes, presents unique challenges in prosthodontic management and rehabilitation. Conventional treatment approaches comprise fixed and removable dental prostheses, which may replace some of the lost structures, but often fall short in fully replacing the impaired oral function and health risks associated with tooth loss. More recent prosthetic treatment modalities comprise implant‐supported fixed and removable prostheses as well as digital manufacturing techniques. However, the extent to which these novel devices restore oral function and enhance patient‐reported outcomes in older adults remains uncertain [10, 11]. Dental prostheses may substantially contribute to the complexity of a restorative case, as they present a number of inherent oral risk factors which need to be monitored [12, 13]. Functional outcomes such as masticatory performance (MP), maximum bite force (MBF), and swallowing are critical indicators of oral function and general well‐being [10]. Likewise, comfort and OHRQoL represent essential patient‐centred dimensions of successful rehabilitation [14, 15, 16]. Despite the growing body of evidence, previous reviews have often been limited to specific prosthetic interventions or patient populations, lacking a comprehensive synthesis that integrates both functional and patient‐reported outcomes across the spectrum of prosthodontic treatments [14, 17, 18].

The aim of this systematic review and meta‐analysis was to assess the effect of the prosthodontic restorations on oral function outcomes and their impact on comfort, denture satisfaction, and OHRQoL in older adults. We address this gap by summarizing the best available evidence and reflecting on the case complexity and risk factors of any given prosthodontic status. The primary outcomes of this systematic review and meta‐analysis were the oral functional outcomes including MP, MBF, and subjectively perceived masticatory ability (MA). Secondary outcomes included OHRQoL, denture satisfaction, and general comfort related to prosthetic rehabilitation.

## 2 Material and Methods

### 2.1 Study Protocol

The present systematic review was performed following the Preferred Reporting Items for Systematic Reviews and Meta‐Analyses [19] and the Cochrane Handbook for Systematic Reviews of Interventions [20]. The study protocol was registered on the PROSPERO International prospective register of systematic reviews and was designed according to the PEO (Population, Exposure, Outcome) framework:

- *Population*: Older adults (Average age: 65 years and above) with tooth loss (partial or complete) wearing any type of dental prostheses for at least 6 months, reporting at least one of the above mentioned functional or patient centred outcomes.
- *Exposure*: Prosthodontic treatment (removable partial or complete conventional dentures (RPD and CD), fixed and removable implant prostheses (IFDP and IOD), and digitally manufactured dentures (DD)).
- *Outcome*: Oral functional outcomes (Masticatory performance; masticatory ability, maximum bite force) as well as Oral Health‐Related Quality of Life (OHRQoL), denture satisfaction, and general comfort.
- *Studies*: Randomized controlled trials, non‐randomized controlled trials, case series, cross‐sectional studies, cohort studies, case–control studies.

Thus, the PEO question was: What is the effect of prosthodontic treatment on the oral function outcomes such as MP and MA, maximum bite force, swallowing function and speech, and the impact on the OHRQoL, denture satisfaction, and comfort in older adults of an age of 65 years or over?

### 2.2 Search Strategy

An electronic search up to August 2025 across four databases: MEDLINE (PubMed), Scopus, Web of Science and the Cochrane Central Register of Controlled Trials (CENTRAL), The search strategy combined controlled vocabulary (MeSH and Emtree terms) with relevant free‐text keywords to enhance sensitivity. No restrictions were applied regarding publication date or language. Full search strategies for each database are provided in Table A1. To ensure completeness, additional manual screening of reference lists from included and relevant studies and reviews was performed. All references were managed using the Rayyan software (Cambridge, MA). A pre‐search was performed together with an expert in research methodology (D.L.) who refined the search to achieve a manageable number of papers. At this stage it was decided to not include a control group, and a PEO framework was adopted for this systematic review.

### 2.3 Eligibility Criteria

Included in this review were studies on humans; with an average age of participants 65 years and above; on individuals with tooth loss (partial or complete) rehabilitated with any type of dental prostheses since at least 6 months; reporting at least one objective and/or subjective functional outcome (e.g., MP, MBF, MA) or OHRQoL, denture satisfaction and general comfort related to prosthetic rehabilitation; with no predefined comparator (although studies including comparison groups were considered for subgroup analysis) and reporting on at least 10 patients per relevant cohort.

Excluded were studies reporting on In vitro or animal studies; including only fully dentate older adults; cohorts with an average age < 65 years; reviews (retained if useful for reference); insufficient documentation on all relevant outcomes; studies where outcome data for prosthesis users cannot be separated from comparison groups; non–peer‐reviewed publication type.

Reports on the same populations, but at different time points, were analysed combined when the same outcome parameters were reported.

### 2.4 Study Selection Process and Data Extraction

**FIGURE 1:**
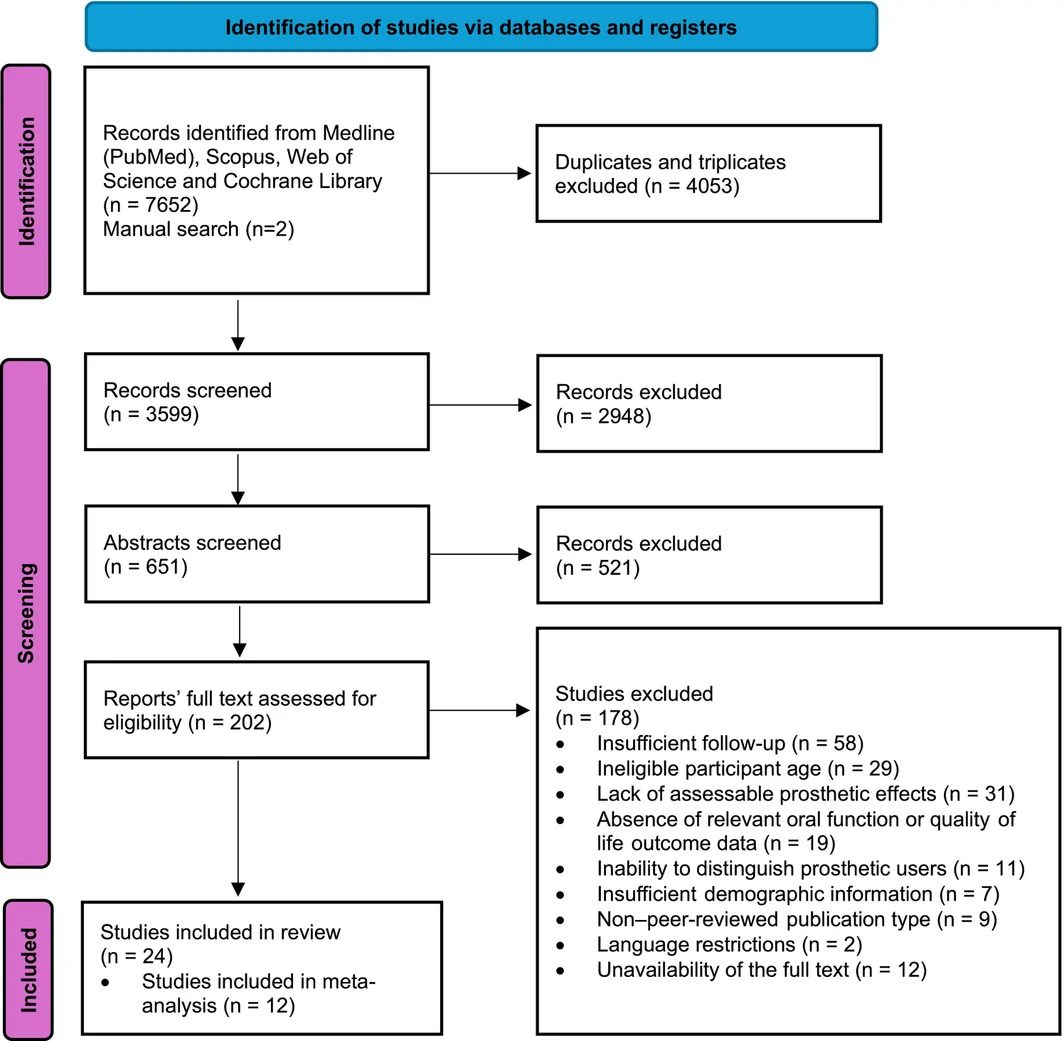
Prisma flow chart. [Colour figure can be viewed at wileyonlinelibrary.com]

Following the database searches, all retrieved records were imported into Rayyan, where duplicates and triplicates were removed prior to screening. Title and abstract screening were independently conducted by three reviewers (A.B., A.N., and P.M.M.) according to predefined eligibility criteria. Records that clearly did not meet the inclusion criteria were excluded at this stage. Full‐text assessment was performed by all reviewers (A.B., N.C., P.M.‐M., M.K., T.O., A.N., M.S., S.T. and F.M.). Consistency of study eligibility decisions was subsequently double‐checked by S.T. and F.M. to ensure agreement across reviewers. Any remaining discrepancies were resolved through group discussion and consensus. The study selection process is summarized in the PRISMA flow diagram (Figure 1).

### 2.5 Risk of Bias

The risk of bias of the included studies was independently assessed by M.K., T.O., M.S., and A.N. using validated tools according to study design. Randomized controlled trials were evaluated using the revised Cochrane Risk of Bias tool (RoB 2), while non‐randomized intervention studies were assessed using the Risk of Bias in Non‐Randomized Studies of Interventions tool (ROBINS‐I, Version 2) [21]. Observational cohort and case–control studies were evaluated using the Newcastle–Ottawa Scale (NOS) according to the original manual, and cross‐sectional studies were appraised using the adapted NOS for cross‐sectional designs [22, 23]. Case series were not included in the risk of bias analysis due to their non‐comparative study design. All risk of bias assessments were conducted in duplicate, with discrepancies resolved through discussion.

### 2.6 Statistical Analysis

According to prosthesis type (implant‐supported vs. non‐implant‐supported) and follow‐up duration (short term: baseline to 6–12 months; long term: baseline to > 12 months), pooled standardized mean differences (SMD) of change scores (post‐intervention minus pre‐intervention) with 95% confidence intervals were estimated using a random‐effects model with the restricted maximum likelihood (REML) estimator. Although analyses were conducted separately by outcome and measurement method, SMDs were used to account for between‐study variability in scale properties and outcome dispersion across heterogeneous study populations. Only outcomes that were measured as pre‐and post in at least two studies were included in the analysis. When the standard deviation (SD) of the change score was not reported, it was calculated from the following formula using a conservative within‐group correlation coefficient of 0.5 where possible.

$${\mathrm{SD}}_{\text{Change}}=\sqrt{{\left({\mathrm{SD}}_{\mathrm{Pre}}\right)}^{2}+{\left({\mathrm{SD}}_{\text{Post}}\right)}^{2}-\left(2\times \rho \times {\mathrm{SD}}_{\mathrm{Pre}}\times {\mathrm{SD}}_{\text{Post}}\right)}.$$

For subjective MA assessed using visual analogue scales (VAS), baseline values were unavailable in several studies. Therefore, pooled mean VAS scores at follow‐up were synthesized as the effect measure, rather than standardized mean differences, to describe post‐treatment masticatory ability. Between‐study heterogeneity was assessed with Cochran's *Q* test and quantified using *I* ^2^ (with *I* ^2^ values of 25%, 50%, and 75% representing low, moderate, and high heterogeneity, respectively). Publication bias was evaluated visually with funnel plots. All statistical tests were two‐sided and a *p* < 0.05 was considered statistically significant. Analyses were performed in STATA (version 17).

## 3 Results

### 3.1 Study Selection

Of the 7652 records initially retrieved from the electronic database search, 4053 were duplicates and removed. 3599 records were screened by title (Figure 1). After the stepwise title and abstract screening, a total of 202 articles were selected for full‐text analysis; full‐text articles were excluded primarily due to insufficient follow‐up duration (*n* = 58), ineligible participant age (*n* = 29), lack of assessable prosthetic effects (*n* = 31), or absence of relevant oral function outcomes or PROMs (*n* = 19). Additional reasons included inability to distinguish prosthetic users (*n* = 11), insufficient demographic information (*n* = 7), non–peer‐reviewed publication type (*n* = 9), language restrictions (*n* = 2), and unavailability of the full text (*n* = 12). Finally, 24 studies met the eligibility criteria and were included in this review.

### 3.2 Description of Included Studies

**FIGURE A1:**
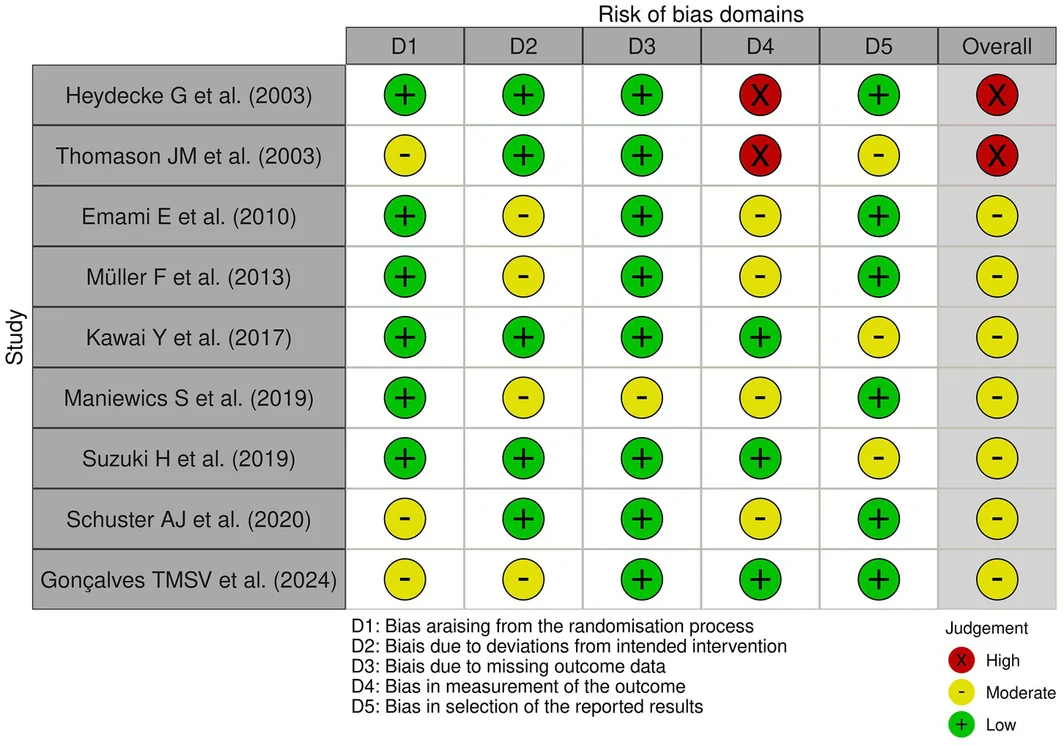
Risk of bias for randomized controlled trials using the revised Cochrane Risk of Bias tool (RoB2). [Colour figure can be viewed at wileyonlinelibrary.com]

**FIGURE A2:**
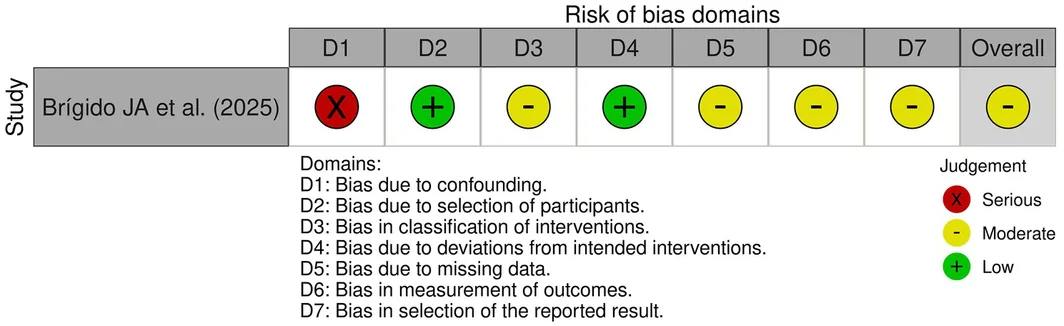
Risk of bias for non‐randomized studies using the ROBINS‐I V2. [Colour figure can be viewed at wileyonlinelibrary.com]

**FIGURE A3:**
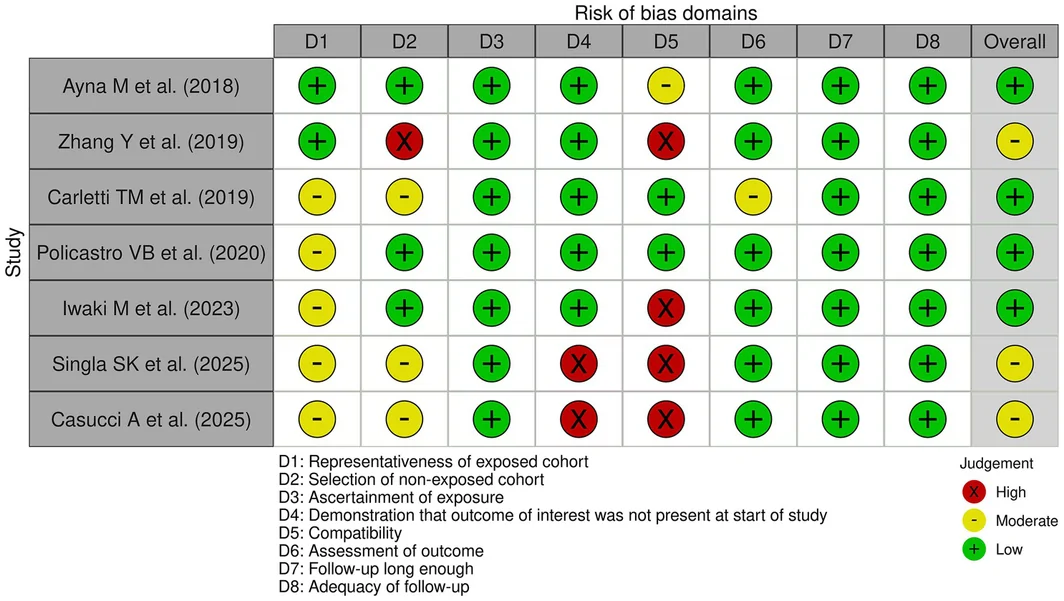
Risk of bias for observational cohort studies using the Newcastle–Ottawa Scale. [Colour figure can be viewed at wileyonlinelibrary.com]

**FIGURE A4:**
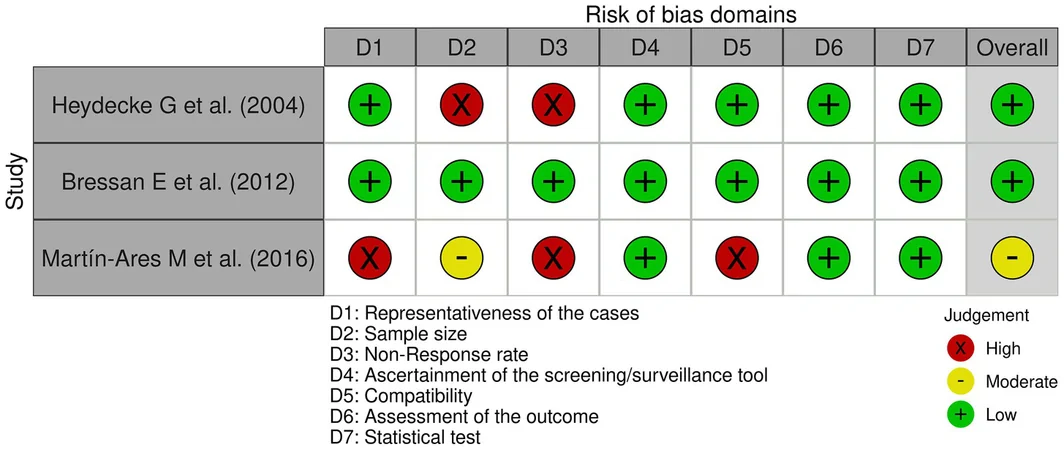
Risk of bias for cross‐sectional designs using the adapted Newcastle‐Ottawa Scale. [Colour figure can be viewed at wileyonlinelibrary.com]

The 24 included publications between 2003 and 2025 comprised 9 randomized controlled trials [22, 23, 24, 25, 26, 27, 28, 29, 30, 31, 32], 1 non‐randomized controlled trial [33], 6 cohort studies [34, 35, 36, 37, 38], 4 cross‐sectional studies [39, 40, 41], 6 case series [42, 43, 44, 45, 46, 47] and included a total number of 1551 patients with mean ages ranging from 65.8 to 85.0 years. Most of the included papers targeted edentulous patients, while only one study reported RPD treatment [43]. Studies were conducted in India, Brazil, Italy, Japan, Canada, Switzerland, Turkey, Spain, Germany, and Hong Kong. The risk of bias is depicted in Figures A1, A2, A3, A4.

### 3.3 Oral Functional Outcomes

Fourteen studies evaluated the effects of prosthetic rehabilitation on oral function outcomes. Four studies compared CDs (*n* = 75) versus IODs (*n* = 73) [28, 29, 32, 34], six studies evaluated CDs alone (*n* = 271) [27, 31, 35, 36, 38, 47] and four IODs alone (*n* = 201) [25, 30, 39, 45]. Overall, oral functional outcomes following CD and IOD rehabilitation showed heterogeneous patterns across studies and outcome measures. Subjective MA generally indicated consistent improvements after treatment, whereas objective MP and MBF showed more variable changes depending on the assessment method, prosthesis type, and follow‐up period.

Nine studies reported on objective MP [25, 28, 29, 30, 31, 35, 37, 38, 45], five on subjective MA [27, 32, 34, 35, 39, 40], and seven on MBF [28, 29, 35, 36, 45, 47]. Objective MP was evaluated with Colour‐mixing Gum (SDHue) in four studies [28, 29, 38, 45], two studies used Colour‐changeable Gum (Xylitol) and gummy Jelly (UHA) [31, 47], two studies used Optocal test food [24, 30], and one used Almond Chewing [35]. Subjective MA was reported in three studies with VAS Scales (100 mm VAS) [27, 31, 34] and two custom‐made Questionnaires [35, 39]. MBF was evaluated in three studies using Occlusal Force‐Meter GM10 [28, 29, 45], one used Gnathodynamometer [36], one used Dental Prescale II, GC [47], and one used Innobyte (Kube Innovation) [38]. Details of each included study are presented in Table 1.

**TABLE 1:** Description of included studies on Oral functional outcomes.

Author	Country	Year	Population characteristics	Study design	Prosthesis type	Age	Sample size	Evaluation periods	Masticatory performance (objective)	Masticatory ability (subjective)	Maximum bite force	Outcome highlight
Thomason et al. [32]	Canada	2003	Community dwelling	RCT	A: IOD (2 implants) B: CD	A: 70.1 (SD 3.2) B: 70.8 (SD 3.0)	A: 30 B: 30	Baseline 6 months	NA	Vas Scale (100mmVAS) Outcomes (BL to 6 M) A: 46 → 82 B: 26 → 64	NA	The IOD group rated the ability to chew significantly higher than the CD group.
Bressan et al. [39]	Italy	2012	Patients at private clinic	Cross‐sectional	IOD (2 implants)	67.7 (range 41–82)	141	The average period of the prosthesis usage: 3.9 years	NA	Questionnaire (Yes or No %) Q1. Can you eat everything? 89.4% Q2. Do you have food impacting under the prostheses? 68.1% Q3. Does the prosthesis move when you chew? 10%	NA	The vast majority reported to be satisfied with the IOD in relation to both functional and aesthetic aspect.
Müller et al. [29]	Switzerland	2013	Care‐dependent older adults	RCT	A: IOD (2 Implants) B: CD with reline	A: 85.0 (SD 6.19) B: 84.1 (SD 5.55)	A: 16 B: 18	Baseline 3 months 12 months	Colour‐mixing Gum (SDHue) Lower values: better mastication (no cut‐off) Outcomes (BL to 12 M): A: 1.49 → 1.33 B: 1.15 → 1.19	NA	Occlusal Force‐Meter GM10 Outcomes (BL to 12 M) A: 44.3 → 73.0 [N] B: 21.2 → 26.4 [N]	Stabilizing existing lower dentures by means of 2 implants in very old care‐dependent edentulous patients provides significantly higher denture satisfaction than conventional reline. The increased maximum bite force also indicates a functional benefit from IOD.
Kawai et al. [27]	Japan	2017	Patients at university dental hospital	RCT	A: CD (lingualized) B: CD (fully bilateral balanced articulation)	A: 72.2 (SD 7.9) B: 72.9 (SD 8.7)	A: 30 B: 30	3 months 6 months	NA	Vas Scale (100mmVAS) Outcomes (3 M to 6 M) A (upper): 94.0 → 95.0 A (lower): 73.5 → 85.0 B (upper): 87.0 → 92.0 B (lower): 75.0 → 76.0	NA	At the 6 month follow up, ratings of satisfaction with fully bilateral balanced articulation mandibular dentures by participants with severe bone resorption revealed significantly lower satisfaction than those with CD with lingualized occlusion.
Carletti et al. [34]	Brazil	2019	Patients at university dental hospital	Cohort Study	A: IOD (1 implant) B: CD	A: 67.7 (SD 4.7) B: 66.5 (SD 7.4)	A: 11 B: 11	6 months	NA	Vas Scale (100mmVAS) Outcomes at 6 M A (upper): 97.8 A (lower): 97.5 B (upper): 93.4 B (lower): 59.5	NA	Wearing a mandibular IOD was more satisfied with their prostheses than patients wearing only conventional CDs, whereas upper satisfaction was similar.
Suzuki et al. [31]	Japan	2019	Patients at university dental hospital	RCT	A: CD with nutrition guidance B: CD without nutrition guidance	A: 74.8 (SD 8.0) B: 78.6 (SD 6.8)	A: 30 B: 29	Baseline 6 months	Colour‐mixing Gum (Xylitol) 0–200 (Higher value: better mastication, about 120 is the general average) Outcome (BL to 6 M) A: 85.1 → 115.5 B: 87.3 → 106.6 Gummy Jelly (UHA) 10‐point (Higher value: better mastication) Outcome (BL to 6 M) A: 2.0 → 4.0 B: 2.0 → 2.0	NA	NA	At each assessment time, there was no significant difference in mixing or shearing ability between groups.
Maniewicz et al. [28]	Switzerland	2019	Care‐dependent older adults	RCT	A: IOD (2 implants) B: CD with reline	A: 85.0 (SD 6.2) B: 84.8 (SD 5.4)	A: 16 B: 16	Baseline Early follow‐up (12 M or earlier) Late follow‐up (12 M or later)	Colour‐mixing Gum (SDHue) Lower values: better mastication (no cut‐off) Outcome (BL to Early to Late) A: 0.79 → 0.73 → 0.66 B: 0.86 → 0.83 → 0.80	NA	Occlusal Force‐Meter GM10 Outcome (BL to Early to Late) MBF overall A: 37.2 → 111.3 → 145.9 [N] B: 57.8 → 48.1 → 72.5 [N]	There was no change in chewing efficiency, whereas the effect of the IODs on the participants' overall bite force was significantly larger than CDs.
Enkling et al. [45]	Switzerland	2019	Patients at university dental hospital	Prospective case series	IOD (4 one‐piece implants)	66.5 (SD 12)	20	Baseline 1 year 5 years	Colour‐mixing Gum (SDHue) Lower values: better mastication (no cut‐off) Outcome (BL → 1Y → 5Y) 0.7825 → 0.8595 → 0.6581	NA	Occlusal Force‐Meter GM10 Outcome (BL → 1Y → 5Y) 58.1 → 112.5 → 150.1 [N]	Chewing efficiency did not change over the first year but was improved at 5 years, while the bite force increased continuously over time.
Policastro et al. [35]	Brazil	2020	Patients at university dental hospital	Prospective Cohort Study	A: CD for normal ridge B: CD for resorbed ridge	66.5 (SD 10.2) A: 64 (SD 2.5) B: 69 (SD 6.8)	A: 14 B: 14	24 h 1 month 3 months 6 months	Almond Chewing Outcome at 6 M A: 30.3% B: 12.4%	Questionnaire (0: unsatisfied, 1: Regular, 2: good) Outcome at 6 M A: 1.64 B: 1.36	Gnathodynamometer (Numerical data was not reported)	The participants with normal ridges have higher masticatory performance and satisfaction with their CD, regardless of the follow‐up period after the insertion of the new CD.
Schuster et al. [30]	Brazil	2020	Patients at university dental hospital	RCT	A: IOD (immediate loading) B: IOD (conventional loading) both 2 Implants	A: 66.8 B: 67.0	A: 10 B: 10	Baseline 1 month 3 months 12 months	Optocal test food Lower values: better mastication (no cut‐off) Outcome: MP_X50 (BL to 12 M) A: 5.04 → 3.31 B: 5.49 → 3.70 Outcome: MPB (BL to 12 M) A: 3.42 → 2.71 B: 4.51 → 3.02	NA	NA	No significant differences were observed between the groups for any of the evaluated periods. However, a statistically significant improvement was observed in masticatory performance after IMO installation, by around 48% for conventional loading and by 44% for immediate loading.
Iwaki et al. [47]	Japan	2024	Patients at university dental hospital	Prospective case series	DCD with digital custom disk method	77.6 (range: 63–87)	20	Baseline 1 month 6 months	Colour‐mixing Gum (Xylitol) 10‐point (Higher value: better mastication) Outcome (BL to 6 M) 6.1 → 7.5 Gummy Jelly (UHA) 10‐point (Higher value: better mastication) Outcome (BL to 6 M) 1.4 → 3.3 Gummy jelly 2 (GC) Higher values: better mastication Outcome (BL to 6 M) Gummy jelly 2: 91.6 → 150.7 (mg/dl)	NA	Dental Prescale II; GC Outcome (BL → 6 M) 290.6 → 263.0 [N]	Significant improvement in masticatory function was observed upon evaluation with gummy jelly 1 & 2, whereas no difference in gum and bite force.
Goncalves et al. [25]	Brazil	2024	Patients at university dental hospital	RCT	A: IOD (4 implants) B: IOD (2 implants)	A: 72.9 (SD 8.4) B: 65.4 (SD 9.6)	A: 10 B: 10	Baseline 6 months 12 months 48 months	Optocal test food Lower values: better mastication (no cut‐off) Outcome (at Baseline, CD) A: 5.35 B: 5.61 Outcome (6 M to 12 M to 48 M, IOD) A: 4.72 → 4.73 → 4.59 B: 4.64 → 4.37 → 4.20	NA	NA	Masticatory performance significantly improved after mandibular IOD installation in both groups. No significant differences were observed between groups with regards to masticatory performance.
Singla et al. [36]	India	2025	Patients at university dental hospital	Cohort Study	A: CD with liner B: CD without liner	A: 67.8 (SD 4.2) B: 66.1 (SD 5.2)	A: 22 B: 22	6 months	NA	NA	Gnathodynamometer Outcome at 6 M A: 66.34 [N] B: 49.41 [N]	The use of soft liners significantly increased maximum bite force over 6 months compared with CD without liners.
Cassucci et al. [38]	Italy	2025	Patients at university dental hospital	Cohort Study	A: CD B: DCD (digital, milling) C: DCD (digital, print)	Total: 74.5 (SD 8.0)	A: 30 B: 15 C: 15	6 months	Colour‐mixing Gum (SDHue) Lower values: better mastication (no cut‐off) Outcome at 6 M A: 0.46 B: 0.47 C: 0.48	NA	Innobyte (Kube Innovation) Outcome at 6 M A: 273.7 [N] B: 269.8 [N] C: 274.9 [N]	No statistically significant differences were observed in bite force or masticatory performance between the groups.

#### 3.3.1 Complete Dentures Functional Outcomes

Regarding the objective outcomes, a colour‐mixing Gum, reported at 6‐months follow‐up, SDHue values (lower value means better MP with no cut‐off) ranged from 0.46 to 0.48 among both conventional and digital CDs [38]. Colour‐changeable Gum (Xylitol), with a digital image analysis (value: 0–200, about 91.4 (male) and 78.0 (female) are the general average) [48], showed improvement from 86.2 at baseline to 111.1 after 6 months on average [31]. In another study using the same method with the 10‐point visual scale also showed improvement from 6.1 to 7.5 over 6 months, confirming enhanced mixing ability consistent with the digital image analysis report. Studies with the MP evaluation with Gummy Jelly, using either the 10‐point visual scale (UHA) and the glucose extraction measurement (GC), reported the improvement by both conventional and digital CDs intervention [47]. MBF evaluation after 6‐months using gnathodynamometer47 demonstrated moderate masticatory strength, achieving a mean bite force from 49.4 to 66.3 N, reflecting a functional biting capacity [36].

Subjective MA evaluated by VAS Scale demonstrated overall improvement from 3 to 6 months. In upper dentures, the mean VAS increased from 90.5 to 93.5 mm, reflecting an improved level of chewing satisfaction, whereas for lower dentures, mean scores increased from 74.3 to 80.5 mm, indicating improvement in perceived MA compared with upper arch [27]. Upper dentures scored consistently better than lower dentures. A subjective Satisfaction Questionnaire, at 6 months, mean scored 1.5 on a scale from 0: unsatisfied, 1: regular, 2: good, corresponding between regular and good. Overall, participants reported the improvement in subjective MA and functional satisfaction [35].

#### 3.3.2 Implant Overdentures Functional Outcomes

Oral function outcomes of IODs included studies on 2 and 4 dental implants. Colour‐mixing Gum method, on 4 one‐piece IODs, showed SDHue values of 0.78 at baseline, increased slightly at 1 year (0.86), and then decreased to 0.66 at 5 years, indicating better colour mixing and enhanced mastication over the long term [45]. The Optocal test food showed a particle size improvement over 12 months, decreasing from 5.04 to 3.31; 3.42 to 2.71 (immediate loading) and from 5.49 to 3.70; 4.51 to 3.02 (late loading), indicating improving comminution regardless of loading protocol [30]. When comparing implant number, baseline Optocal values with CDs were similar (4‐implant: 5.35; 2‐implant: 5.61). After delivery, both groups showed values decreased from 4.72 to 4.73 to 4.59 (4‐implant) and from 4.64 to 4.37 to 4.20 (2‐implant) over 6, 12, and 48 months [25]. Subjective MA (functional questionnaire) showed favourable experiences; 89.4% reported that they could eat everything; however, 68.1% indicated experiencing food impaction beneath the prosthesis, and 10% reported that their prosthesis moved during chewing [39]. MBF on 4‐implant IODs increased substantially over time, from 58.1 N at baseline to 112.5 N at 1 year, reaching 150.1 N at 5 years [45].

#### 3.3.3 Complete Denture Versus Implant Overdentures Functional Outcomes

Regarding the studies comparing the Oral function outcomes CDs versus IODs, the MP assessment using Colour‐mixingGum (SDHue) showed a modest improvement from baseline to 12 months, changing from 1.49 to 1.33 in IODs and 1.15 to 1.19 in CDs [29]. Longitudinal analysis from the same patient cohort demonstrated a further gradual improvement, with SDHue values decreasing from 0.79 IODs; 0.86 CDs at baseline to 0.73; 0.83 (early) and 0.66; 0.80 (late), respectively [28]. Subjective MA (VAS Scale) improved in both groups, with greater gains observed for two implant IODs. From baseline to 6 months, VAS scores increased from 46 to 82 mm in the IODs and from 26 to 64 mm in the CDs [32]. For one mandibular implant IODs, at 6 months, arch‐specific scores showed high satisfaction (upper: 97.8 mm; lower: 97.5 mm), whereas CDs achieved high ratings only in the upper arch (93.4 mm) and lower satisfaction in the antagonist arch (59.5 mm) [34]. MBF (Occlusal Force‐Meter GM10) 2‐implant IODs, increased from 44.3 to 73.0 N over 12 months, compared with CDs (21.2 to 26.4 N) [29]. MBF increased sharply with 2‐implant overdentures (37.2 to 111.3 to 145.9 N), while complete dentures showed only modest change (57.8 to 48.1 to 72.5 N) [28].

### 3.4 OHRQoL and Other Patient Reported Outcome Measures

Eighteen studies evaluated the effect of prosthetic rehabilitation on OHRQoL. Five studies compared CDs (*n* = 165) versus IODs (*n* = 184) [5, 26, 30, 32, 34], one study compared IODs (*n* = 16) versus fixed full‐arch prostheses (*n* = 16) [33] and one study CDs (*n* = 50) vs IODs (*n* = 50) versus fixed full‐arch prostheses (*n* = 50) [41]. Five studies evaluated CDs (*n* = 460) [27, 35, 39, 40, 44, 49], four IODs (*n* = 306) [30, 37, 39, 46], one fixed full‐arch implant prostheses (*n* = 15) [42] and one RPDs (*n* = 44) [43]. The OHRQoL was based on the OHIP‐14 in five studies [26, 33, 38, 43, 45], two studies the OHIP‐20 [24, 26], two studies the OHIP‐Edent [29, 30, 42] and one study reported OHIP‐14 and OIDP [42], the OHIP‐16 [27] and the Modified GOHAI‐11 [44]. Other Patient Reported Outcomes were included in three studies using VAS Scale (100mmVAS) for denture satisfaction and/or comfort [27, 28, 32, 34], three custom‐made satisfaction questionnaires for aesthetics and function [35, 39] and one Modified OHIP‐14 (9 questions) [41]. Details of each included study are presented in Table 2.

**TABLE 2:** Description of included studies on oral health‐related quality of life and patient reported outcomes.

Author	Country	Year	Population characteristics	Study design	Prosthesis type	Age	Sample size	Evaluation periods	OHRQoL total score	Other patient reported outcome	Outcome highlight
Thomason et al. [32]	Canada	2003	Community dwelling	RCT	A: IOD (2 implants) B: CD	A: 70.1 (SD 3.2) B: 70.8 (SD 3.0)	A: 30 B: 30	Baseline 6 months	NA	Vas Scale (100 mmVAS) General satisfaction (BL to 6 M) A: 34 → 81 B: 32 → 59 Comfort (BL to 6 M) A: 34 → 86 B: 26 → 63	General satisfaction ratings were higher in the IOD than in the CD by approximately 36%.
Heydecke et al. [26]	Germany	2003	Community dwelling	RCT	A: IOD (two implant) B: CD	A: 68.9 (SD 3.2) B: 69.4 (SD 2.7)	A: 30 B: 30	Baseline 2 months 6 months	OHIP‐20 (BL to 6 M) 0–80 (Lower values: better OHRQoL) A: 53.5 → 35.0 B: 56.3 → 47.8	OHIP‐20 (Subdomain score range was not reported) Psychological discomfort (BL to 6 M) A: 5.3 → 3.3 B: 6.0 → 4.6 Physical pain (BL to 6 M): A: 14.0 → 8.1 B: 15.5 → 12.4	Results for the IOD were also superior in the functional limitation, physical pain, physical disability and psychological disability subscales.
Heydecke et al. [32]	Germany	2004	Community dwelling	Cross‐sectional	CD	66.0 (SD 12.0)	249	The average period of the prosthesis usage: 3.2 year	OHIP‐14 0–56 (Lower values: better OHRQoL) 27.3	OHIP‐14 Subdomain (0–8: lower = better) Psychological discomfort: 4.0 Physical pain: 4.9	Wearing CDs has a significant impact on OHRQOL. This impact is moderated by the styles a patient uses to cope with stress. Using emotional support has a positive effect on OHRQOL.
Berretin‐Felix et al. [42]	Brazil	2008	Patient at university dental hospital	Case series	Fixed full‐arch prosthesis (implants) (5 implant)	66 (range 60–76)	15	Baseline 3 months 6 months 18 months	OHIP‐14 (BL to 3 M to 6 M to 18 M) 0–56 (Lower values: better OHRQoL) 18 → 6 → 0 → 3 OIDP (BL to 3 M to 6 M to 18 M) 0–100 (%) 20 → 0 → 0 → 0	NA	Scores in the OIDP and OHIP‐14 questionnaires were better after IOD intervention.
Emami et al. [5]	Canada	2010	Patient at university dental hospital	RCT	A: IOD (2 Implants) B: CD	72.1 (SD 4.4)	173 A: 97 B: 76	12 months (no BL data)	OHIP‐20 at 12 M 0–80 (Lower values: better OHRQoL) A: 31.83 B: 38.55	NA	There was no significant difference in OHIP scores for males and females or socio‐demographic groups except for marital status. Married participants had statistically significant lower OHIP scores than single or separated participants.
Geckili et al. [46]	Turkey	2011	Patient at university dental hospital	Case series	IOD (2‐implants)	65–82	Total: 78	Baseline 6 months	OHIP‐14 (BL to 6 M) 0–56 (Lower values: better OHRQoL) 31.8 → 8.4	OHIP‐14 Subdomain (0–8: lower = better) Psychological discomfort (BL to 6 M) 6.3 → 2.4 Physical pain (BL to 6 M): 6.7 → 1.7	Compared to pretreatment scores, subjects had statistically significant decreases in total and all subscale scores of OHIP‐14 after receiving IOD.
Bressan et al. [39]	Italy	2012	Patients at a private clinic	Cross‐sectional study	IOD (2 implants)	67.7 (range 41–82)	141	The average period of the prosthesis usage: 3.9 years	NA	A questionnaire (Yes/No, %) Satisfaction for aesthetic 98% satisfied Satisfaction for function 98% satisfied	The vast majority of patients reported to be satisfied in relation to the restorative therapy from both functional and aesthetic points of view.
Müller et al. [29]	Switzerland	2013	Care‐dependent older adults	RCT	A: IOD (2 Implants) B: CD with reline	A: 85.0 (SD 6.19) B: 84.1 (SD 5.55)	A: 16 B: 18	Baseline 3 months 12 months	OHIP‐EDENT (BL to 12 M) 0–80 (Lower values: better OHRQoL) A: 41.1 → 21.9 B: 32.9 → 23.4	Vas Scale (100mmVAS) 24 predefined questions (range: 0–2400) Denture Satisfaction (BL → 12 M) A: 749.4 → 1687.9 B: 948.7 → 1145.3	The IOD showed an improved oral health–related quality of life than the CD with reline.
Dable et al. [44]	India	2013	Patient at university dental hospital	Case series	CD	69.4 (range 60–82)	63	Baseline 6 months	Modified GOHAI‐11 (BL to 6 M) the 12th item was excluded which was related to the sensitivity of teeth 28.9 → 42.2	NA	There was a significant change in the quality of life in elders after CD rehabilitation, though the self‐rated general health did not show any significant improvement.
Martin‐Ares et al. [41]	Spain	2016	Patient at university dental hospital	Cross‐sectional study	A: CD B: Fixed full‐arch prosthesis (implants) C: IOD (4 implnats)	72 (range 66–80)	A: 50 B: 50 C: 50	The average period of the prosthesis usage: at least 5 years	NA	Modified OHIP‐14 (9 questions) at 1Y (each question: 0–4: lower = better) Pain in mouth: A: 2.50 B: 0.66 C: 0.90 Discomfort eating: A: 2.04 B: 0.64 C: 0.84 Function disability A: 0.00 B: 0.02 C: 0.06	Only 14% of patients with CD experienced almost complete satisfaction, compared to 36% of patients with IOD and 46% of patients with fixed prostheses.
Kawai et al. [27]	Japan	2017	Patients at university dental hospital	RCT	A: CD (lingualized) B: CD (fully bilateral balanced articulation)	A: 72.2 (SD 7.9) B: 72.9 (SD 8.7)	A: 30 B: 30	3 months 6 months	OHIP‐16 (3 M to 6 M) 0–64 (Lower values: better OHRQoL) A: 20 → 9.5 B: 21.5 → 7.0	Vas Scale (100mmVAS) Comfort (3 M → 6 M) A (upper): 95 → 97 A (lower): 75 → 96 B (upper): 91 → 86 B (lower): 75 → 75	Participants with severe bone resorption and fully bilateral balanced CD rated their satisfaction with retention of mandibular dentures significantly lower than those with lingualized CD. They also had significantly lower OHRQoL for the domain of Pain.
Ayna et al. [33]	Germany	2018	Patients at university dental hospital	Non‐RCT	A: IOD (4 implants) B: Fixed full‐arch prosthesis (implants)	A: 71.5 (SD 3.9) B: 72.1 (SD 4.4)	A: 16 B: 16	Baseline 1 year 3 years 5 years 7 years	OHIP‐14 (BL to 1Y to 3Y to 5Y to 7Y) 0–56 (Lower values: better OHRQoL) A: 24.6 → 0.6 → 1.3 → 2.1 → 2.6 B: 31.2 → 0.2 → 0.8 → 1.6 → 2.2	NA	There was a dramatic subjective improvement in both groups. There were no differences in the OHIP scores between patients with bar‐retained and ceramic dentures.
Carletti et al. [34]	Brazil	2019	Patients at university dental hospital	Cohort study	A: IOD (1 implant) B: CD	A: 67.7 (SD 4.7) B: 66.5 (SD 7.4)	A: 11 B: 11	6 months (No BL data)	NA	Vas Scale (100 mmVAS) General Satisfaction (at 6 M) A: 98.3 B: 84.9 Comfort (at 6 M) A (upper): 98.9 A (lower): 97.0 B (upper): 90 B (lower): 63.2	Patients in the CD group were less content than the IOD group in all satisfaction categories in the VAS.
Zhang et al. [37]	Hong Kong	2019	Patients at university dental hospital	Cohort study	IOD	71.3 years	67	Baseline 6 months 1 year 2 years 3 years 4 years 5 years	NA	Questionnaire (6 questions) 5‐point scale/each (0: very satisfied, 4: very dissatisfied) Overall Satisfaction (at BL, 6 M (IOD), 1Y, 2Y, 3Y, 4Y and 5Y) 7.00 → 5.00 → 4.50 → 2.00 → 1.00 → 1.00 → 1.00	Mandibular IOD was a beneficial treatment option for seniors with history of deficient CD, improving denture quality, patient satisfaction, and reducing patient complaints up to 5 years
Policastro et al. [35]	Brazil	2020	Patients at university dental hospital	Cohort study	A: CD for normal ridge B: CD for resorbed ridge	A: 64.0 (SD 2.5) B: 69.0 (SD 6.8)	A: 14 B: 14	24 h 1 month 3 months 6 months	NA	Questionnaire (0: unsatisfied, 1: Regular, 2: good) General Satisfaction (at 6 M) A: 1.86 B: 1.50 Comfort (upper/lower) A (upper): 2.00 A (lower): 1.86 B (upper): 1.79 B (lower): 1.29	The participants with normal ridges have higher satisfaction with their CD, regardless of the follow‐up period after the insertion of the new CD.
Schuster et al. [30]	Brazil	2020	Patients at university dental hospital	RCT	A: IOD (immediate loading) B: IOD (conventional loading) both 2 Implants	A: 66.8 B: 67.0	A: 10 B: 10	Baseline 1 month 3 months 12 months	OHIP‐EDENT (BL to 3 M to 6 M) 0–80 (Lower values: better OHRQoL) A: 18.5 → 1.0 → 1.0 B: 5.0 → 1.0 → 1.0	NA	Differences were observed only between before and after the IOD treatment intervention, in the physical pain, physical disability, handicap, and global score.
Cassucci et al. [38]	Italy	2025	Patients at university dental hospital	Cohort study	A: CD B: DCD (digital, milling) C: DCD (digital, print)	74.5 (SD 8.0)	A: 30 B: 15 C: 15	6 months	OHIP‐14 at 6 M 0–56 (Lower values: better OHRQoL) A: 16.63 B + C: 16.53 *B&C cannot be separated.	NA	OHIP‐14 scores indicated slightly lower patient satisfaction with DCD, but the difference was not clinically significant.
Brígido et al. [43]	Brazil	2025	Type 2 diabetes mellitus Patients at university dental hospital	Case series	RPD (details not specified)	65.8 (range 60 to 83)	44	Baseline 12 months	OHIP‐14 0–56 (Lower values: better OHRQoL) 6.3 → 2.7	OHIP‐14 Subdomain (0–8: lower = better) Psychological discomfort (BL to 12 M) 1.0 → 0.5 Physical pain (BL to 12 M) 1.0 → 0.7	OHRQoL significantly improved after prosthodontic therapy combined with dietary advice among older adults with type 2 diabetes.

Across studies, improvements in OHRQoL were generally observed following prosthodontic rehabilitation; however, the magnitude and consistency of change varied according to prosthesis type, outcome measure, and follow‐up duration. Studies involving implant‐supported prostheses, particularly IODs, more frequently demonstrated greater improvements in OHRQoL and patient satisfaction than conventional CDs, although not all studies reported clinically meaningful between‐group differences.

#### 3.4.1 Complete Denture Versus Implant Overdentures OHRQoL

OHRQoL improved more in 2 implants IODs where OHIP‐20 decreased from 53.5 to 35.0 at 6 months, compared with 56.3 to 47.8 in CDs [26]. At 12 months, IODs maintained slightly better outcomes 31.8 than CDs 38.6 [5]. OHIP‐EDENT improved in both groups over 12 months, with a greater reduction in oral‐health impact scores in 2 implant IODs (41.1 to 21.9) compared to CDs (32.9 to 23.4) [29]. Subjective Outcomes using VAS 10 mm reported greater satisfaction and comfort for IODs. From baseline to 6 months, general satisfaction increased from 34 to 81 mm in IODs and 32 to 59 mm in CDs. Comfort improved from 34 to 86 mm in the IODs and 26 to 63 mm in the CDs [32]. Psychological discomfort decreased more with IODs than with CDs (IOD 5.3 to 3.3; CD 6.0 to 4.6). Physical pain showed improvement (IOD 14.0 to 8.1; CD 15.5 to 12.4) [26]. General Satisfaction VAS Scale (100 mmVAS) at 6 months remained higher for IODs (98.3 mm) than CDs (84.9 mm). IODs showed superior comfort in both arches (upper: 98.9 mm; lower: 97.0 mm) compared with CDs (upper: 90.0 mm; lower: 63.2 mm) [34]. A 24‐item VAS 100 mm satisfaction score increased more in IODs 749.4 to 1687.9 than in CDs from 948.7 to 1145.3, at 6 months, IODs showed higher satisfaction 98.3 mm and comfort in both arches [29].

#### 3.4.2 Implant Overdentures Versus Fixed Full‐Arch Prostheses OHRQoL

OHIP‐14 scores for IODs fell from 24.6 at baseline to 0.6 at 1 year, rising slightly to 2.6 at 7 years. Fixed full‐arch implant prostheses showed similar improvement, decreasing from 31.2 to 0.2 at 1 year and reaching 2.2 at 7 years [31].

#### 3.4.3 Complete Denture Versus Implant Overdentures Versus Fixed Full‐Arch Prostheses OHRQoL

At 1‐year, modified OHIP‐14, patients with CDs reported higher impacts than those with implant restorations. Pain was greatest in CDs (2.50) compared with fixed prostheses (0.66) and IODs (0.90). Discomfort showed the same pattern (2.04 vs. 0.64 vs. 0.84). Functional disability remained low, implants‐showed minimal impacts (0.02–0.06) [41].

#### 3.4.4 Complete Dentures OHRQoL

At baseline, CDs reported a mean OHIP‐14 sum score of 27.3. Subdomain analyses showed psychological discomfort (4.0) and physical pain (4.9) [40]. At 6 months, conventional CDs showed 16.6 versus 16.5 for digitally manufactured dentures [38], indicating a comparable reduction in oral‐health impacts. Modified GOHAI‐11 increased from 28.9 to 42.2 after 6 months, indicating better perceived oral health [44]. CDs baseline OHIP‐16 ranged from 20.0 to 21.5, decreasing to 7.0–9.5 at 6 months [28]. The comfort of CDs improved (100 mm VAS Scale) from 91–95 mm at 3 months to 86–97 mm at 6 months in upper dentures and from 75 mm at 3 months to 75–96 mm at 6 months for lower dentures [27]. At 6 months, CD satisfaction ranged from 1.5 to 1.9 (scale 0 to 2). Comfort was higher for upper dentures (1.8–2.0) than for lower ones (1.3–1.9) [35].

#### 3.4.5 Implant Overdentures OHRQoL

The OHIP‐14 for IODs led to large improvements in OHRQoL, decreasing from 31.8 to 8.4 by 6 months. OHIP‐EDENT scores improved in immediate loading from 18.5, 1.0 to 1.0 (baseline, 3, 6 months) and in conventional loading from 5.0, 1.0 to 1.0, indicating minimal oral‐health impact [30]. IODs showed reductions in OHIP‐14 subdomains, with psychological discomfort improving from 6.3 to 2.4 and physical pain from 6.7 to 1.7 by 6 months [46]. Patient satisfaction was high, with 98% of patients being satisfied with aesthetics and 98% with function [35]. Overall satisfaction improved from 7.00 at baseline to 5.00 at 6 months, 4.50 at 1 year, and levelled at 1.00 from 3 to 5 years [37].

#### 3.4.6 Fixed Full‐Arch Implant Prostheses OHRQoL

OHIP‐14 scores declined from 18 at baseline to 6, 0 and 3 by 3, 6, and 18 months. OIDP scores dropped from 20% at baseline to 0% during the follow‐up, indicating complete resolution of daily oral impacts [42].

#### 3.4.7 Removable Partial Dentures OHRQoL

OHRQoL improved after RPD treatment, total OHIP‐14 scores decreased from 6.3 to 2.7. Psychological discomfort improved from 1.0 to 0.5, and physical pain from 1.0 to 0.7, indicating fewer oral health impacts over 12 months [43].

### 3.5 Meta‐Analyses

Table 3 presents summary measures of change in the study outcomes (post‐intervention minus pre‐intervention) within the study groups (implant‐supported prostheses and non‐implant‐supported prostheses), according to the follow‐up period (6–12 months; > 12 months). Furthermore, forest plots and funnel plots corresponding to each outcome are provided in the Appendix A of the manuscript.

**TABLE 3:** Summary of pooled within‐group changes and post‐treatment outcomes following prosthodontic rehabilitation in older adults.

Outcome	Group	Follow‐up	*N*	Pooled effect size		Heterogeneity	
				SMD (95% CI)	*p*	*I* ^2^	*p*
Objective masticatory performance	Implant‐supported prostheses	6–12 m	2	−0.033 (−0.824, 0.759)	0.935	82.90%	0.016
	Implant‐supported prostheses	> 12 m	2	−0.601 (−0.945, −0.256)	< 0.001	0%	0.730
Maximum bite force	Implant‐supported prostheses	6–12 m	2	0.668 (0.142, 1.193)	0.013	55.44%	0.134
	Implant‐supported prostheses	> 12 m	2	1.124 (0.719, 1.529)	< 0.001	0%	0.662
Subjective masticatory ability^a^	Implant‐supported prostheses	6–12 m	2	90.571 (75.304, 105.838)	< 0.001	89.69%	0.002
	Non‐implant supported prostheses	6–12 m	3	76.700 (64.558, 88.842)	< 0.001	94%	< 0.001
Oral health impact profile—14	Implant‐supported prostheses	6–12 m	3	−2.550 (−4.637, −0.463)	0.017	96.93%	< 0.001
	Implant‐supported prostheses	> 12 m	2	−2.612 (−6.250, 1.026)	0.159	97.02%	< 0.001
Oral health impact profile—20	Implant‐supported prostheses	6–12 m	2	−1.007 (−1.358, −0.656)	< 0.001	0%	0.517
	Non‐implant supported prostheses	6–12 m	3	−0.948 (−2.007, 0.111)	0.079	89.61%	< 0.001

Abbreviations: CI, confidence interval; *N*, number of studies; SMD, standardized mean difference of change.

^a^ Due to lack of baseline values, only mean VAS score at the follow‐up was estimated as the effect size.

#### 3.5.1 Objective MP (Colour‐Changeable Gum Colour Method, SDHue)

For SDHue, the pooled analysis in implant‐supported prostheses showed no significant change in the short term (SMD = −0.03, 95% CI: −0.82 to 0.75; *p* = 0.93), accompanied by substantial heterogeneity (*I* ^2^ = 82.9%). In contrast, a significant reduction in SDHue was observed in the long term (SMD = −0.60, 95% CI: −0.94 to −0.25; *p* < 0.001), with no detectable heterogeneity (*I* ^2^ = 0%). These findings suggest improvements in SDHue may become more evident over longer follow‐up periods (Figures A5a, A5b, A5c, A5d).

#### 3.5.2 MBF Force (Occlusal Force‐Meter, GM10)

In implant‐supported prostheses, MBF showed a significant increase both in the short term (SMD = 0.66, 95% CI: 0.14 to 1.19; *p* = 0.01) and long term (SMD = 1.12, 95% CI: 0.71 to 1.52; *p* < 0.001). Moderate heterogeneity was observed in the short‐term analysis (*I* ^2^ = 55.4%), while the long‐term results were highly consistent across studies (*I* ^2^ = 0%). Overall, these results indicate a sustained and clinically meaningful improvement in MBF over time following treatment (Figures A6a, A6b, A6c, A6d).

#### 3.5.3 Subjective MA (VAS Scale)

Subjective MA assessed by VAS scale; significant improvements were observed in both groups in the short‐term. Because baseline values were unavailable in several studies, standardized mean differences could not be calculated. Therefore, pooled mean VAS scores at follow‐up were synthesized as the effect measure. In implant‐supported prostheses, the pooled mean VAS was markedly reduced (Mean = 90.5, 95% CI: 75.3 to 105.8; *p* < 0.001), although heterogeneity was considerable (*I* ^2^ = 89.7%). Similarly, non‐implant supported prostheses demonstrated a significant reduction (Mean = 76.7, 95% CI: 64.5 to 88.8; *p* < 0.001), also with high heterogeneity (*I* ^2^ = 94%). These findings should be interpreted with caution, as baseline values were unavailable and only mean follow‐up scores were synthesized (Figures A7a, A7b, A7c, A7d).

#### 3.5.4 OHIP–14

Implant‐supported prostheses showed a significant improvement in OHIP‐14 at short‐term follow‐up (SMD = −2.55, 95% CI: −4.63 to −0.46; *p* = 0.017), indicating a reduction in OHRQoL impairment. However, this effect was accompanied by substantial heterogeneity across studies (*I* ^2^ = 96.9%). At long‐term follow‐up, although the pooled estimate suggested a continued improvement, the effect did not reach statistical significance (SMD = −2.61, 95% CI: −6.25 to 1.02 *p* = 0.15), with similarly high heterogeneity (*I* ^2^ = 97%). These findings suggest that while short‐term improvements in OHIP‐14 may be evident following implant treatment, the long‐term effect remains uncertain due to marked between‐study variability or few number of studies (Figures A8a, A8b, A8c, A8d).

#### 3.5.5 OHIP–20

For the OHIP‐20, a significant improvement was observed in the implant‐supported prostheses group at short‐term (SMD = −1, 95% CI: −1.35 to −0.65; *p* < 0.001), with no observed heterogeneity (*I* ^2^ = 0%). In contrast, the non‐implant supported prostheses showed a non‐significant pooled effect (SMD = −0.94, 95% CI: −2 to 0.1; *p* = 0.07), accompanied by substantial heterogeneity (*I* ^2^ = 89.6%). These results suggest a more consistent improvement in oral health–related quality of life when implants are used (Figures A9a, A9b, A9c, A9d).

## 4 Discussion

This systematic review was undertaken to evaluate the functional and patient‐reported outcomes in old and very old persons with tooth loss that had been treated with any type of removable denture at least 6 months prior to the examination. This review demonstrates that among functional outcomes, MBF shows the most consistent and sustained improvement following prosthodontic rehabilitation, particularly with implant‐supported prostheses, whereas objective MP improves more gradually and subjective outcomes show substantial variability. These improvements were observed across different prosthesis types and were largely independent of fabrication method, with no consistent differences between digitally manufactured and conventionally fabricated dentures [36]. Compared to CDs, MBF increases substantially and consistently in implant‐supported prostheses. Conventional denture performance is heavily influenced by anatomical conditions, such as ridge resorption [35] but also the presence of liners [36]. MBF is lower in older patients [45]. Objective MP improves more gradually, although it did not improve in a very old cohort where the denture teeth were not replaced along with the implant insertion [29]. These findings suggest that age‐related decline may limit objective performance gains while leaving perceived function largely preserved. The patient‐reported outcome measures were in general positive, with most VAS satisfaction scores well on the positive or very positive side of the scale [27, 34] and various OHRQoL and satisfaction indices improving during the adaptation period [37]. Patients judge IODs more favourably than CDs, but depending on conditions, the latter can also be very satisfactory, especially in the upper jaw [27, 32, 34]. Most problems were reported for the lower conventional denture [27, 34]. The frequency and severity of complaints is higher in the first 6 months after denture insertion, but technical complications and other adverse events are not uncommon for longer periods [37].

A limitation of this review is that most included analyses were non‐comparative and focused on within‐group changes over time, without a consistent control group, which precludes direct comparisons between prosthetic modalities and should not be interpreted as demonstrating superiority. However, there is a huge body of evidence, especially in younger adult populations, that all types of dental prostheses fall short in fully restoring chewing function and OHRQoL [6, 50]. For this reason, the present systematic review focuses on patients with an age of 65 years or over, and a PEO framework (population, exposure, outcome) was adopted. Where meta‐analyses included only two studies, pooled estimates mainly reflect consistency in direction of effect rather than precise quantification.

A strength of this review is that it focuses rather on age and the effect of tooth loss than the type of restoration; hence, all types of dentures were included to provide a robust estimation of what restorative care can or cannot provide in terms of functional and patient reported outcomes.

Old and very old persons were chosen for this review, as age affects several characteristics of our chewing behaviour. Whereas the number of cycles needed to chew a standard piece of food increases progressively with age, the masticatory frequency, the shape and the amplitude of the chewing cycle remain unchanged [51]. The capacity to adapt to changes in the hardness of food seems maintained [52]. Tooth loss, although not a physiological effect of old age, accumulates along with the sequelae of dental diseases over a lifetime and further reduces the masticatory efficiency [50]. Other factors that determine MP are the chewing muscle strength, oral mechanoreception and the patient's motor‐coordination, all of which deteriorate with age. A reduced number of occlusal units and tooth wear may further degrade the MP in old age, although a shortened dental arch with 20 remaining teeth is considered an acceptable compromise [53]. Tooth loss, along with an impaired MP, may be considered a risk factor for muscle atrophy and malnutrition [54, 55]. Last but not least, enjoying meals, preferably in the company of others, is one of the highlights in the day of many old persons and an essential part of their quality of life. Hence the functional restoration of a partially or fully edentulous person by means of removable prosthesis is of utmost complexity and importance.

To effectively revert masticatory impairment by prosthodontic means, retention and stability are critical factors. The patients perceive masticatory function as easier with fixed implant dental prostheses; furthermore, the functional limitation dimension of OHRQoL measures is significantly better than in complete denture wearers [56]. Conventional complete dentures are seated on the edentulous alveolar ridge, and their function is assured by physical retention, occlusion, and muscle coordination from the lips, cheek, and the tongue, all of which deteriorate with age. Along with progressive alveolar ridge resorption, denture stability and therefore MP proved compromised. Policastro and collaborators found a 2.5 times larger particle size in patients with advanced ridge resorption after 40 chewing cycles of a standard size silicone cube [35]. MP is frequently limited by pain on the denture bearing tissues and fear of dislodgement of the denture body. Implant overdentures are an effective means to provide not only denture retention, but also mechanical support, and can be considered beneficial for the denture performance at large and MP in particular. A geriatric study on dependent elders could however not confirm this effect, probably as at the very advanced age and reduced general health status of the cohort the improvement by the implants was overshadowed by the age‐related decline in function [29]. Furthermore, in this study, the worn denture teeth were not replaced along with implant placement, which may have further hindered an improvement in MP. Several studies from this review consistently confirmed an increasing MBF after the insertion of implant dentures [28, 29]. MBF is further dependent on the atrophy of the alveolar ridges, but also on the denture wearing habits as well as the sensitivity of the underlying denture bearing tissues [49]. MBF relates further to the cross‐sectional area of the masseter muscle, which is smaller in women than in men.

Several studies revealed that functional improvement with removable dentures is not immediate and refer to a period of at least 6 months until the outcome parameter change levels out [25, 27, 29, 30, 31, 32, 47]. This adaptation phase is probably caused by the re‐organization of neuronal circuits in the Central Nervous System to adapt motor patterns for chewing, as well as speech and other oral functions. The adaptation to new or replacement dentures may stretch over months and can be monitored by fMRI imaging [57]. The concept of “neuroplastic prosthodontics” addresses this issue and provides clinical recommendations to facilitate adaptation [58]. Other more mechanical adaptation processes concern the settling of the denture into the denture bearing tissues. An (initial) tissue soreness may explain why dentures with liners showed higher bite forces than those with heat‐polymerized intaglio surfaces [36] which may alleviate soreness but at the same time may present mechanical and hygienic challenges. During this initial stage after denture insertion, the patient would benefit from psychological guidance and clinical support.

The review identified objective measures for MP as well as subjectively perceived MA. Clinical test methods like the sieving method (particle size analysis of a test specimen), colour changeable gums (Xylitol), colour mixing ability (SDHue) or glucose extraction techniques (UHA) provide objective measures for the MP, whereas MA is evaluated subjectively, often using VAS scales. In this review, subjective MA tended to demonstrate larger and more consistently positive changes, whereas objective MP tests showed improvement with smaller magnitudes and greater methodological variability. Subjective MA reflects a patient‐perceived dimension of oral function that is closely related to OHRQoL rather than to objective MP alone. This phenomenon is known as the geriatric paradoxon, which describes an age‐related discrepancy between a normative treatment need and the treatment demand in older adults. Steele analysed statistically the effect of age on the OHRQoL in edentulous patients and reported an improvement with age [59]. An improvement of the general self‐reported well‐being beyond the age of 50 years would confirm this paradoxon [60]. The results indicate that in a clinical context, it is important to verify the patients' subjective views with objective measures of oral function.

Interpretation of OHRQoL outcomes, particularly those synthesized using SMDs, requires caution. Due to the wide score ranges and relatively small standard deviations of OHIP instruments, SMD values may appear numerically large and should not be interpreted using conventional Cohen thresholds.

Although the review confirms that OHRQoL improves over the months following denture insertion, some dimensions tend to remain impaired. The high heterogeneity reflects the clinical diversity of older populations and the variability in treatment modalities. The dimensions of physical pain and psychological discomfort improve to an excellent level, but they never decrease to zero, which would represent a non‐compromised OHRQoL [26, 40, 41, 43, 46]. A dental prosthesis is a non‐biological replacement of what from a neurological point of view can be considered as an “amputated organ”. No dental material can replace the tactile sensitivity of the periodontal receptors and fully restore chewing function and even aesthetics. Removable dentures must be removed for cleaning, which some patients might consider a substantial psychological shortcoming. In this context, the OHRQoL scores reported in this review seem very good, and patients seem to cope amazingly well. Implant restorations, fixed or removable, may substantially contribute to improving the OHRQoL of denture wearers and improving their social activities and inclusion [7].

Along with tooth loss and any type of prosthodontic treatment, the complexity of treatment increases, and several risk factors apply. With the day of insertion, biological and technical complications may occur. Zhang and collaborator reported on the frequency and severity of patient complaints and found that both were highest in the first 3 months post insertion [37]. Although the frequency of complaints continued thereafter, they were minor in terms of intensity when compared to the initial complaints. Complaints may relate to pain, denture retention and stability, chewing and speech problems, increased salivary flow, “finding the new occlusion”, foreign body feeling, accidental biting injury, burning mouth sensation or vomiting reflexes and last but not least to aesthetics. In very old patients with reduced neuroplasticity, adaptation may be delayed or fail. Material intolerances may occur with regards to chrome‐cast frameworks or resin dentures. Further to patients' complaints, a regular adaptation of the prosthesis to the aging oral environment is required via relines and/or occlusal adjustments. Finally, patients who have experienced tooth loss might benefit from some sensitive psychological guidance, to respect their prosthetic privacy and potential feeling of embarrassment.

As the currently available body of evidence relates mainly to younger adults, future research should focus on functional impairment and restorative treatments in old and very old age cohorts, as demographic changes indicate an increasing life‐expectancy and tooth retention until later in life.

## 5 Conclusions

In older adults, prosthodontic rehabilitation is associated with improvement in oral function and patient‐reported outcomes, with the most consistent gains observed for maximum bite force following implant‐supported prostheses, particularly over longer follow‐up. Objective masticatory performance appears to improve more gradually, whereas masticatory ability is generally rated as high after treatment regardless of prosthesis type. Improvements in oral health–related quality of life are evident, especially for implant‐supported prostheses, although results vary considerably depending on the outcome measure used and are characterized by substantial heterogeneity. The observed heterogeneity across outcomes likely reflects differences in study methodologies and the clinical diversity of older adult populations, including variations in functional status, prosthetic configurations, and assessment tools. These findings emphasize the importance of follow‐up duration, particularly given the high maintenance needs during the first months after prosthesis insertion and the need for careful interpretation of pooled estimates in this population.

## Author Contributions

**Pedro Molinero‐Mourelle:** methodology, conceptualization, writing – original draft, data curation, writing – review and editing, visualization, investigation. **Azize Bell:** methodology, data curation, writing – review and editing. **Najla Chebib:** methodology, conceptualization, writing – review and editing, validation. **Manabu Kanazawa:** methodology, conceptualization, writing – review and editing, investigation. **Frauke Müller:** conceptualization, methodology, writing – original draft supervision, resources, project administration, investigation. **Annika Neri:** methodology, data curation, writing – review and editing. **Takahiro Ono:** methodology, conceptualization, writing – review and editing, investigation. **Masako Shiramizu:** data curation, writing – review and editing. **Sayaka Tada:** validation, writing – review and editing, writing – original draft, methodology, data curation, visualization, investigation.

## Funding

The authors have nothing to report.

## Ethics Statement

The authors have nothing to report.

## Consent

All authors have approved the submitted version and consent to the publication.

## Conflicts of Interest

The authors declare no conflicts of interest.

## Figures and Tables

**FIGURE A5a:**
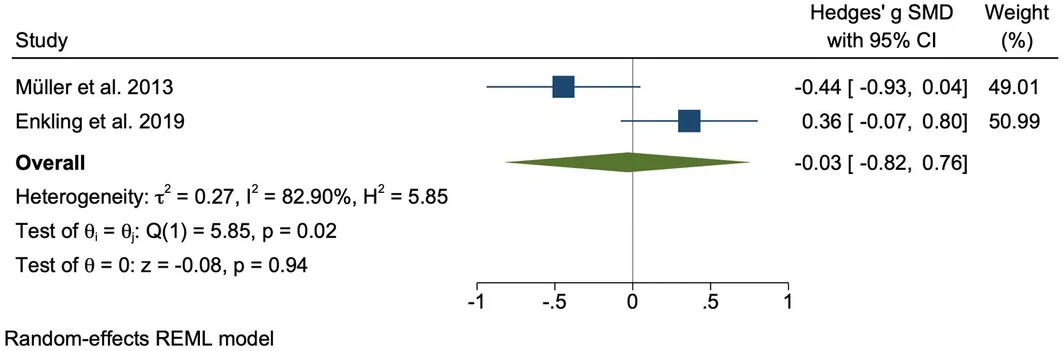
Forest plot presenting SMD of objective masticatory performance (Colour‐mixing Gum colour method, SDHue) of implant‐supported prostheses and follow‐up (6–12 months). [Colour figure can be viewed at wileyonlinelibrary.com]

**FIGURE A5b:**
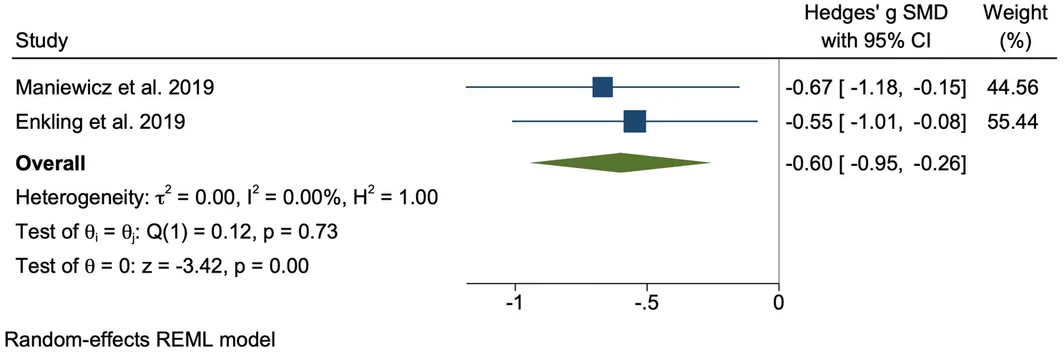
Forest plot presenting SMD of objective masticatory performance (Colour‐mixing Gum colour method, SDHue) of implant‐supported prostheses and follow‐up (> 12 months). [Colour figure can be viewed at wileyonlinelibrary.com]

**FIGURE A5c:**
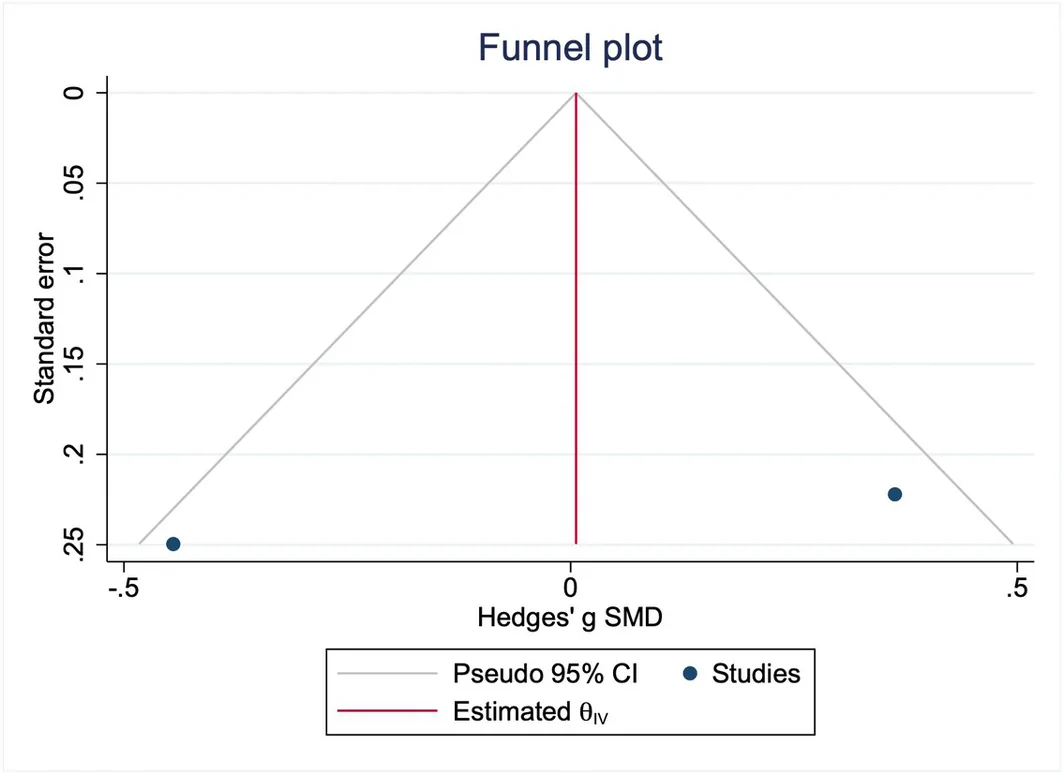
Funnel plot presenting publication bias for objective masticatory performance (Colour‐mixing Gum colour method, SDHue) of implant‐supported prostheses and follow‐up (6–12 months). [Colour figure can be viewed at wileyonlinelibrary.com]

**FIGURE A5d:**
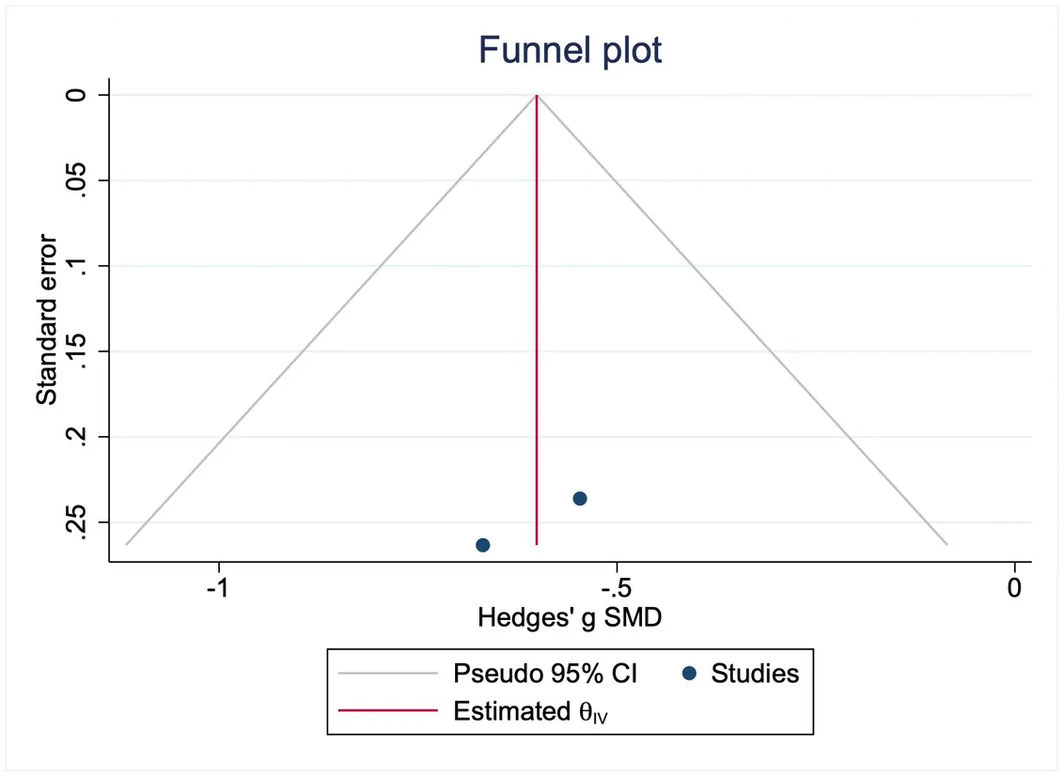
Funnel plot presenting publication bias for objective masticatory performance (Colour‐mixing Gum colour method, SDHue) of implant‐supported prostheses and follow‐up (> 12 months). [Colour figure can be viewed at wileyonlinelibrary.com]

**FIGURE A6a:**
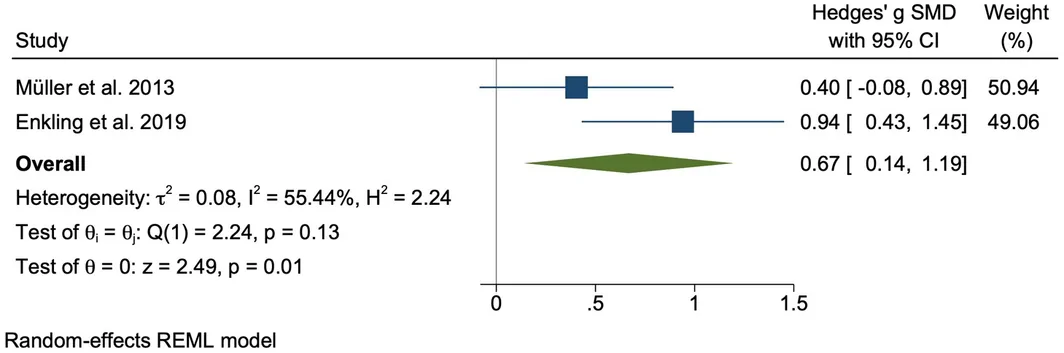
Forest plot presenting SMD of maximum bite force (Occlusal Force‐Meter, GM10) of implant‐supported prostheses and follow‐up (6–12 months). [Colour figure can be viewed at wileyonlinelibrary.com]

**FIGURE A6b:**
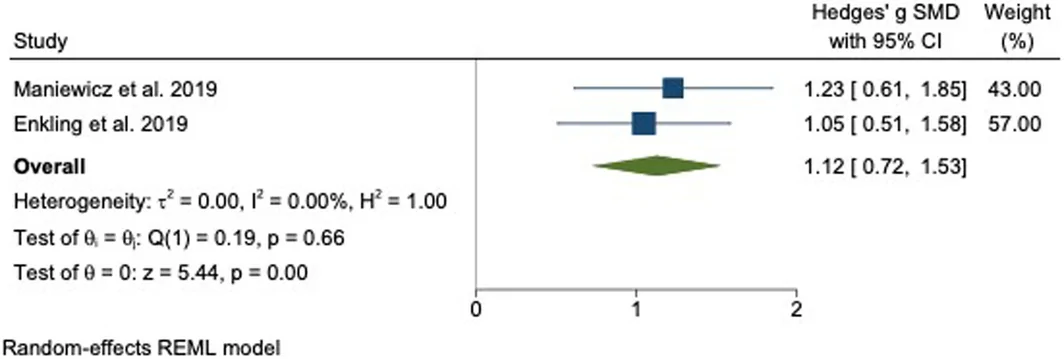
Forest plot presenting SMD of maximum bite force (Occlusal Force‐Meter, GM10) of implant‐supported prostheses and follow‐up (> 12 months). [Colour figure can be viewed at wileyonlinelibrary.com]

**FIGURE A6c:**
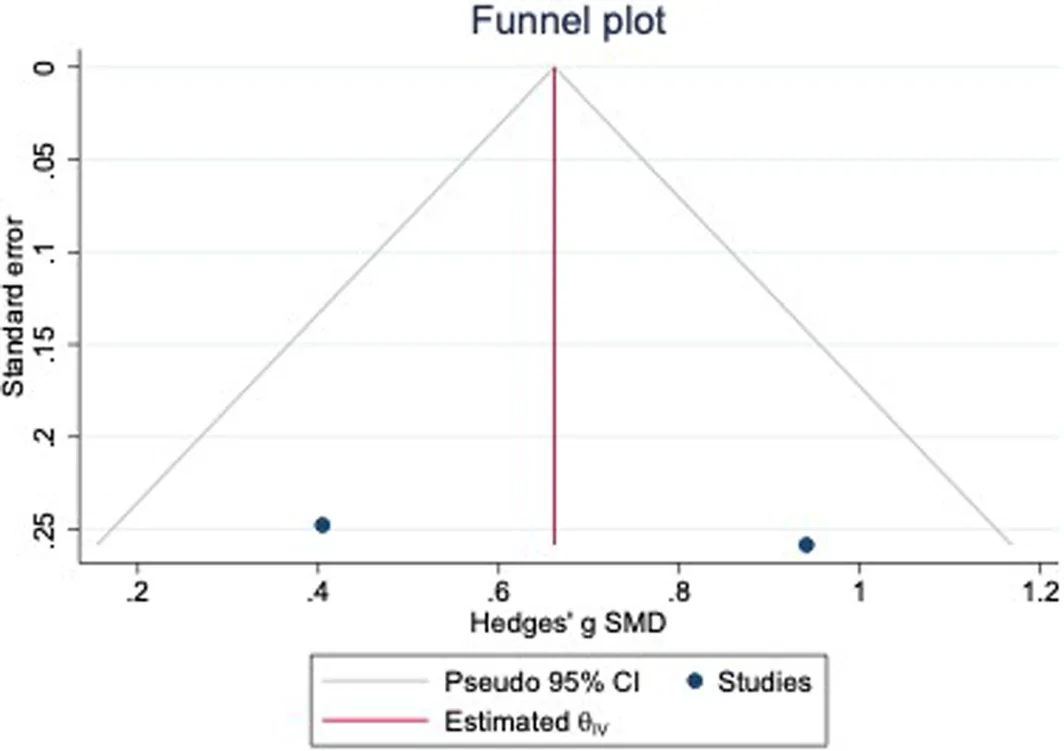
Funnel plot presenting publication bias for maximum bite force (Occlusal Force‐Meter, GM10) of implant‐supported prostheses and follow‐up (6–12 months). [Colour figure can be viewed at wileyonlinelibrary.com]

**FIGURE A6d:**
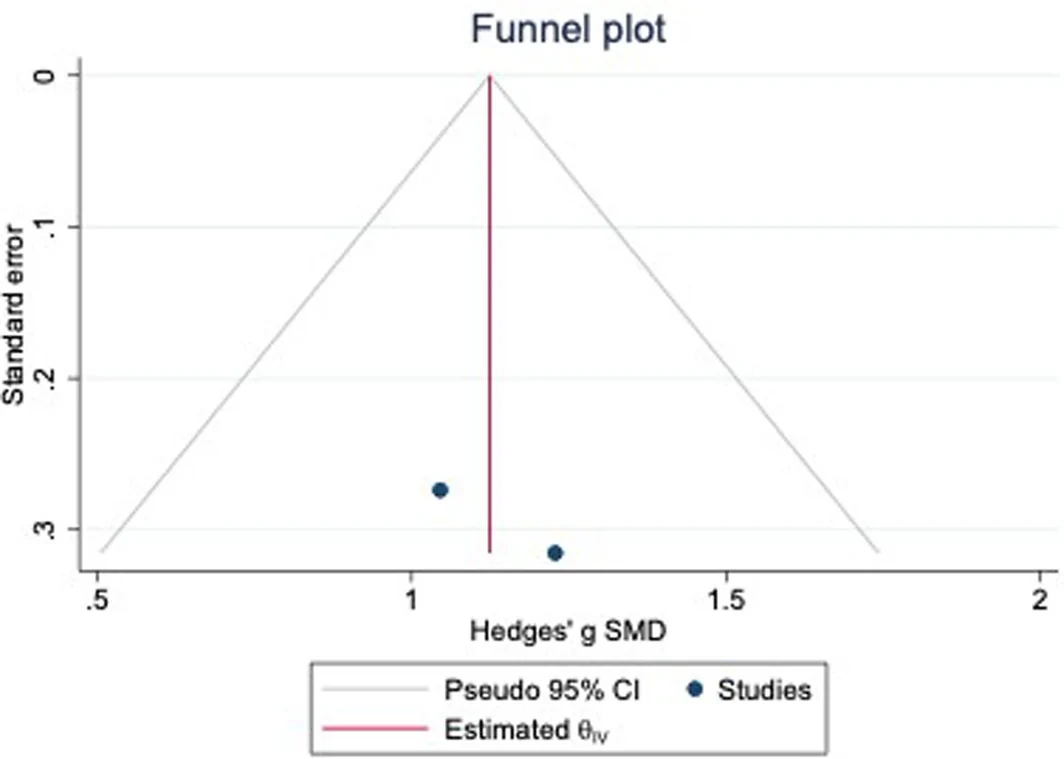
Funnel plot presenting publication bias for maximum bite force (Occlusal Force‐Meter, GM10) of implant‐supported prostheses and follow‐up (> 12 months). [Colour figure can be viewed at wileyonlinelibrary.com]

**FIGURE A7a:**
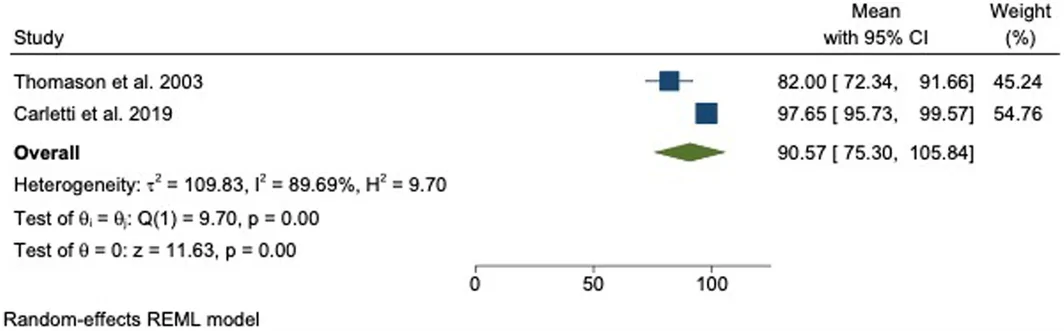
Forest plot presenting Mean of subjective masticatory ability (VAS Scale) of implant‐supported prostheses and follow‐up (6–12 months). [Colour figure can be viewed at wileyonlinelibrary.com]

**FIGURE A7b:**
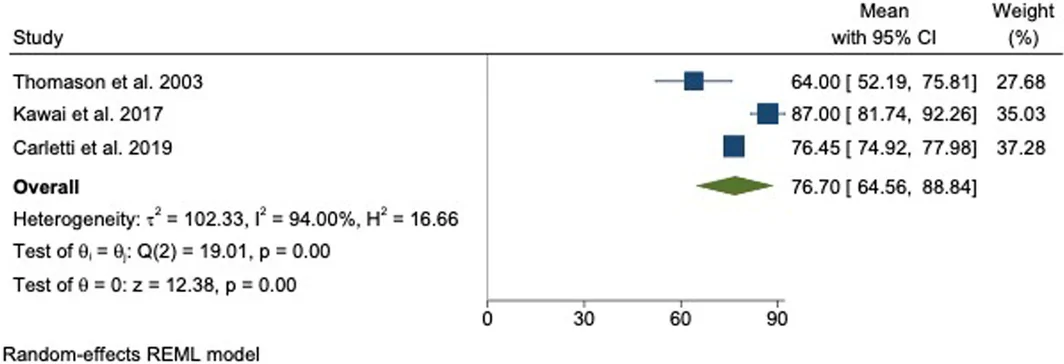
Forest plot presenting mean of subjective masticatory ability (VAS Scale) of non‐implant supported prostheses and follow‐up (6–12 months). [Colour figure can be viewed at wileyonlinelibrary.com]

**FIGURE A7c:**
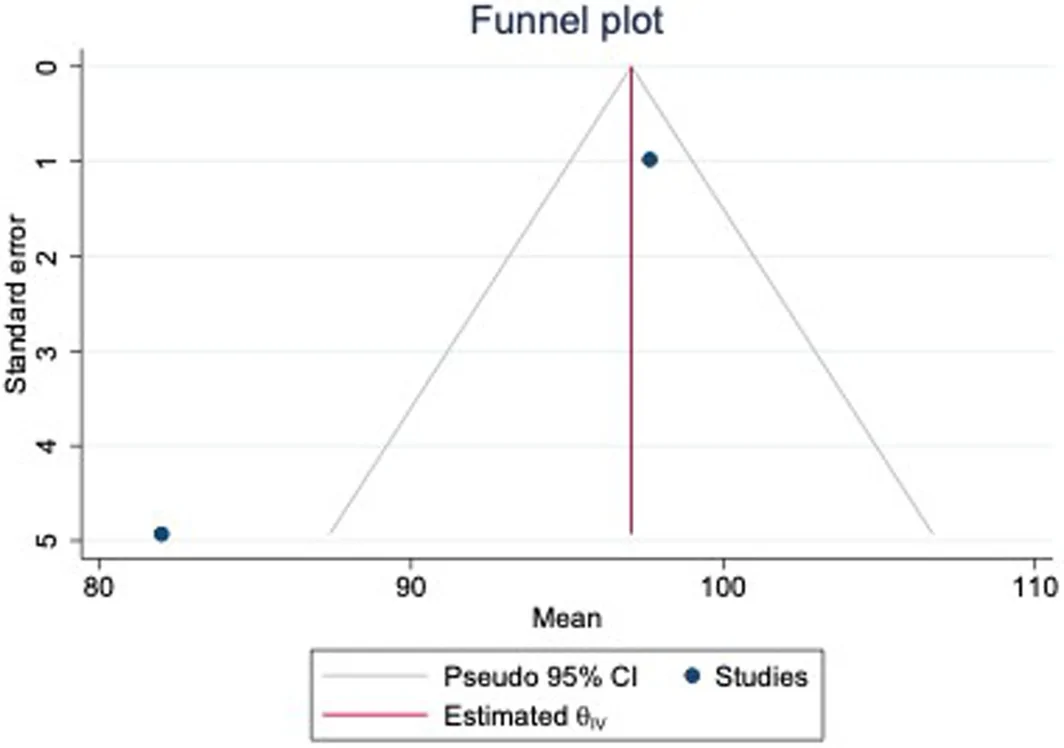
Funnel plot presenting publication bias for subjective masticatory ability (VAS Scale) of implant‐supported prostheses and follow‐up (6–12 months). [Colour figure can be viewed at wileyonlinelibrary.com]

**FIGURE A7d:**
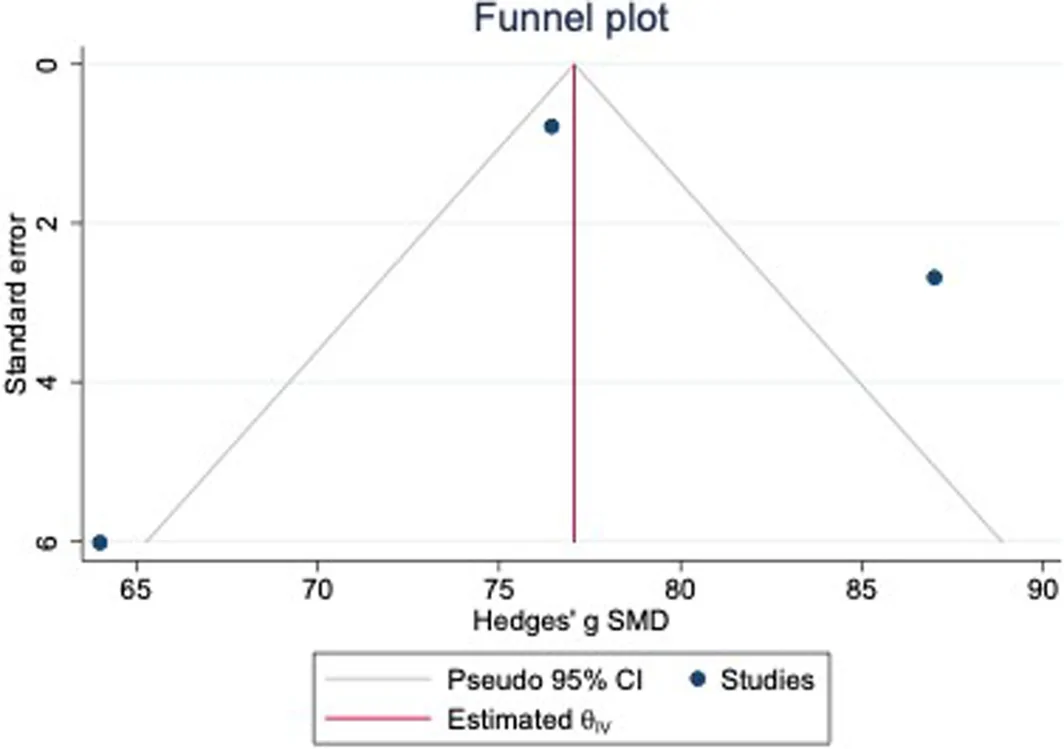
Funnel plot presenting publication bias for subjective masticatory ability (VAS Scale) of non‐implant supported prostheses and follow‐up (6–12 months). [Colour figure can be viewed at wileyonlinelibrary.com]

**FIGURE A8a:**
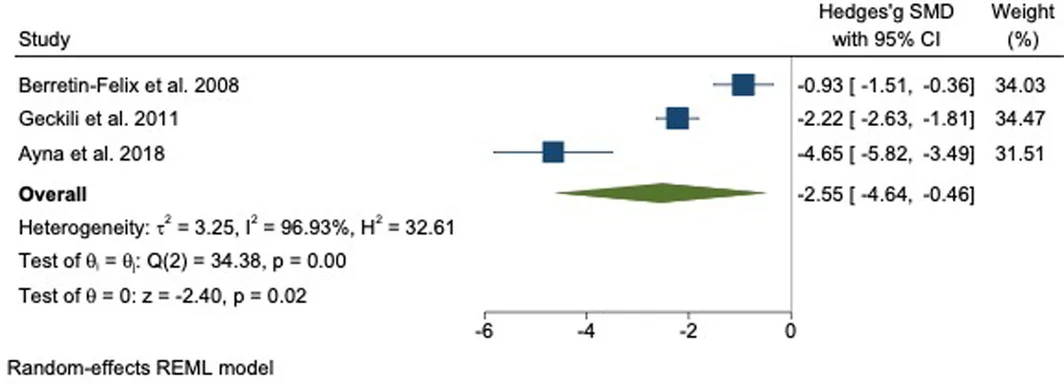
Forest plot presenting SMD of OHIP‐14 for implant‐supported prostheses and follow‐up (6–12 months). [Colour figure can be viewed at wileyonlinelibrary.com]

**FIGURE A8b:**
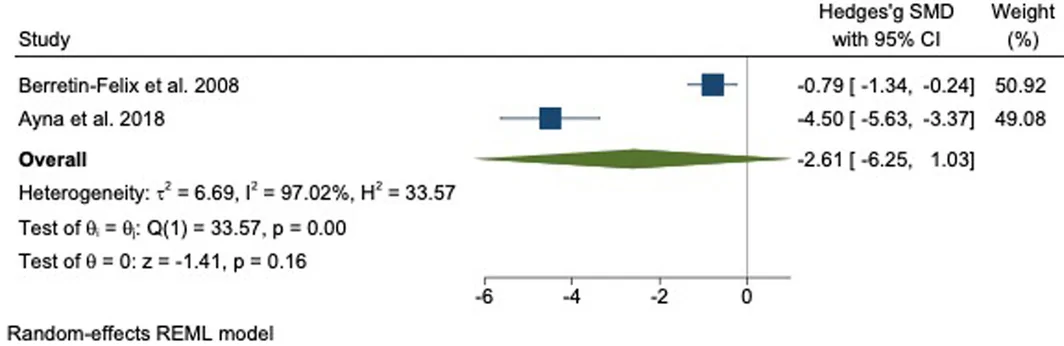
Forest plot presenting SMD of OHIP‐14 for implant‐supported prostheses and follow‐up (> 12 months). [Colour figure can be viewed at wileyonlinelibrary.com]

**FIGURE A8c:**
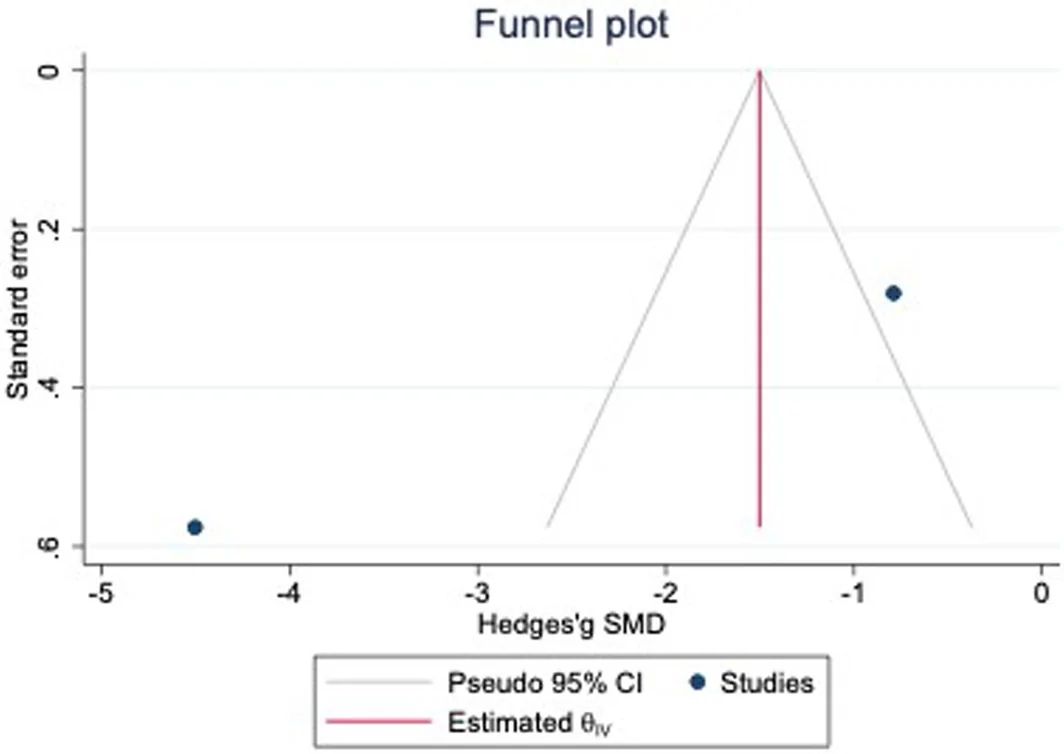
Funnel plot presenting publication bias of OHIP‐14 for implant‐supported prostheses and follow‐up (6–12 months). [Colour figure can be viewed at wileyonlinelibrary.com]

**FIGURE A8d:**
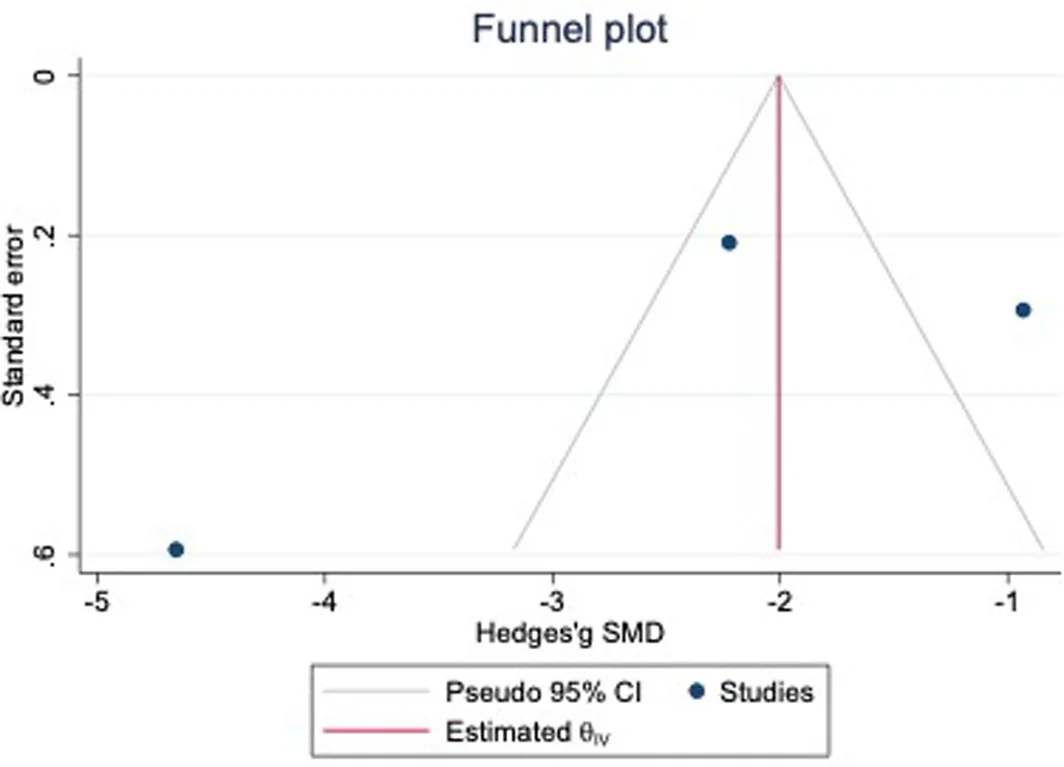
Funnel plot presenting publication of OHIP‐14 for implant‐supported prostheses and follow‐up (> 12 months). [Colour figure can be viewed at wileyonlinelibrary.com]

**FIGURE A9a:**
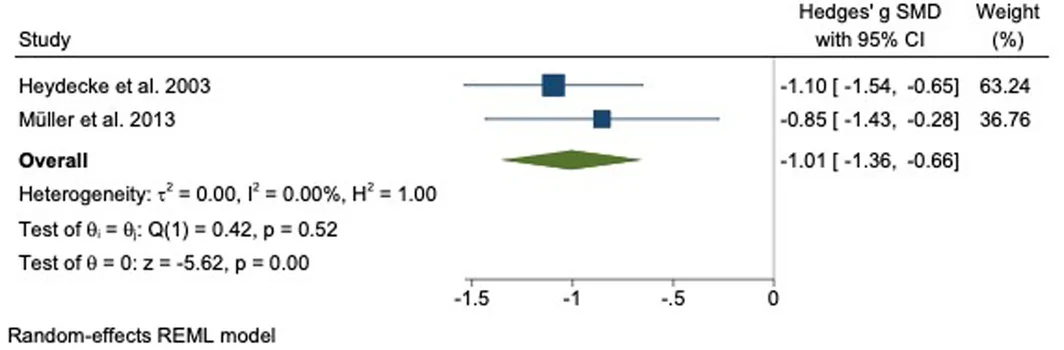
Forest plot presenting SMD of OHIP‐20 for implant‐supported prostheses and follow‐up (6–12 months). [Colour figure can be viewed at wileyonlinelibrary.com]

**FIGURE A9b:**
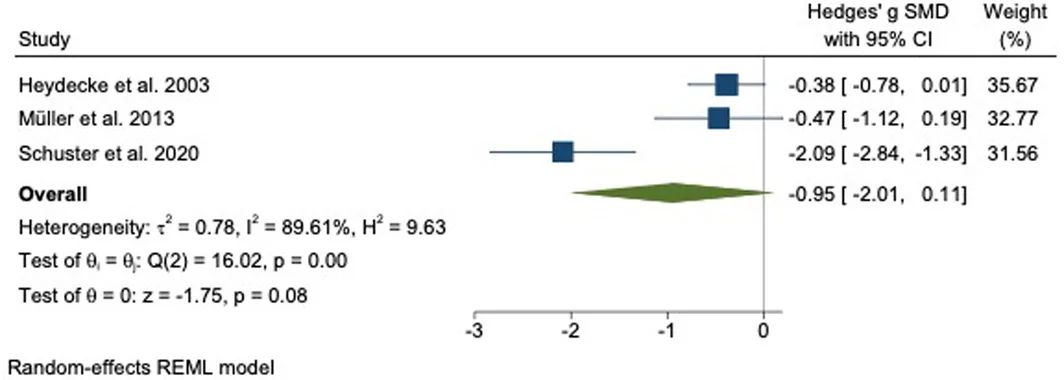
Forest plot presenting SMD of OHIP‐20 for non‐implant supported prostheses and follow‐up (> 12 months). [Colour figure can be viewed at wileyonlinelibrary.com]

**FIGURE A9c:**
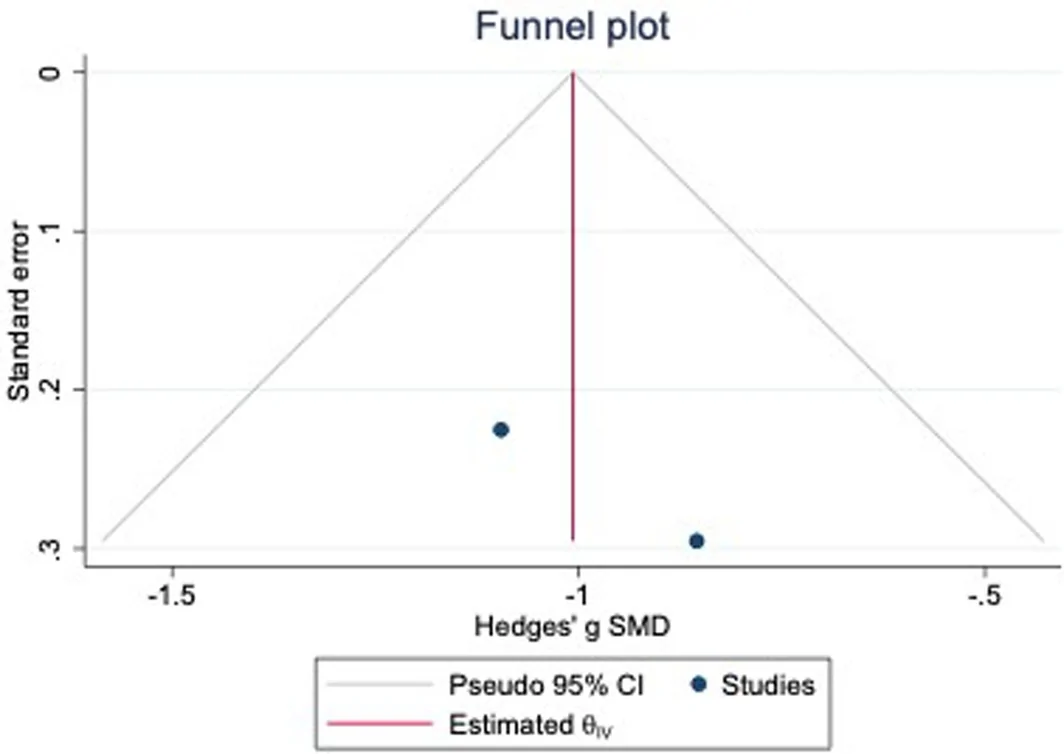
Funnel plot presenting publication bias of OHIP‐20 for implant‐supported prostheses and follow‐up (6–12 months). [Colour figure can be viewed at wileyonlinelibrary.com]

**FIGURE A9d:**
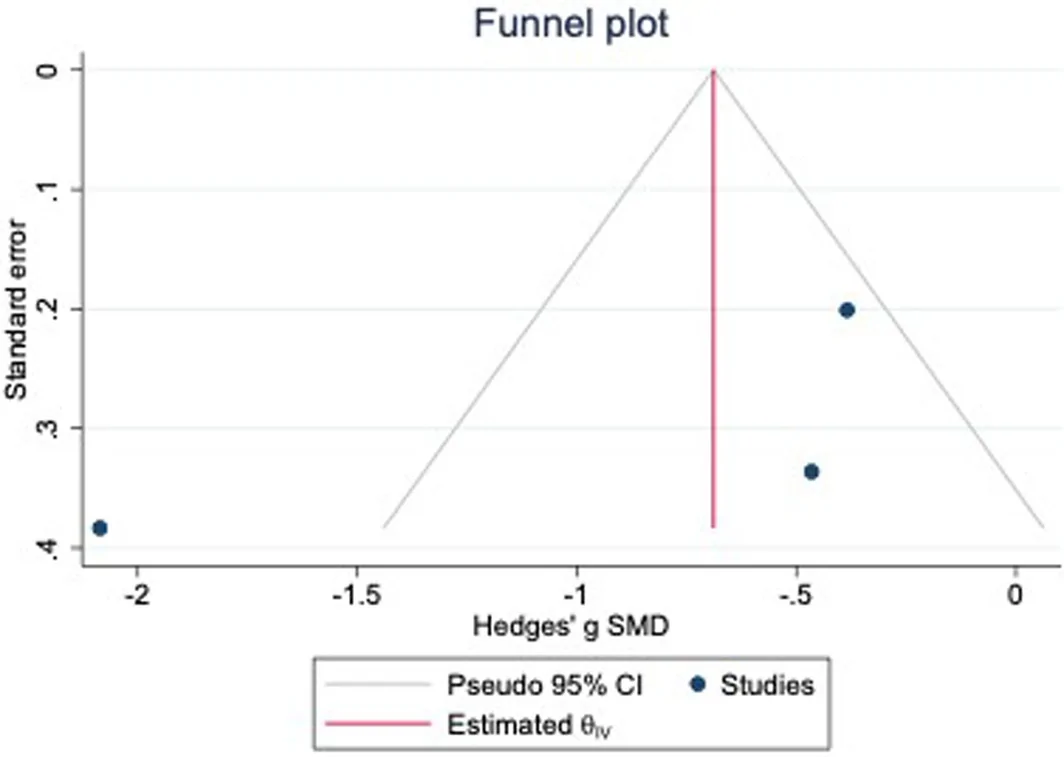
Funnel plot presenting publication bias of OHIP‐20 for non‐implant supported prostheses and follow‐up (> 12 months). [Colour figure can be viewed at wileyonlinelibrary.com]

## Data Availability

The data that support the findings of this study are available on request from the corresponding authors. The data are not publicly available due to privacy or ethical restrictions.

## Appendix A

**TABLE A1:** Systematic search strategies applied in different databases according to the PEO research framework.

Focused question	What is the effect of the prosthodontic treatment on the oral function outcomes such as masticatory performance and ability, maximum bite force, swallowing function and speech and the impact on the OHRQoL, denture satisfaction and comfort in older adults of an age of 65 years or over?	
Search strategy	PubMed	(“Elder*”[Title/Abstract] OR “Geriatric Population*”[Title/Abstract] OR “Geriatric Patient*”[Title/Abstract] OR “Aging Population”[Title/Abstract] OR “Aged Population”[Title/Abstract] OR “Aged 60”[Title/Abstract] OR “60 year*”[Title/Abstract] OR “Gerodont*”[Title/Abstract] OR “Aged 75”[Title/Abstract] OR “75 year*”[Title/Abstract] OR “Aged 80”[Title/Abstract] OR “80 year*”[Title/Abstract] OR “Senior*”[Title/Abstract] OR “Care‐depend*”[Title/Abstract] OR “Dental Care for Aged”[MeSH Terms] OR “Older Adults”[Title/Abstract] OR “Older People” [Title/Abstract]) AND (“Tooth Loss”[MeSH Terms] OR “tooth loss”[Title/Abstract] OR “edent*”[Title/Abstract] OR “missing teeth”[Title/Abstract] OR “Jaw, Edentulous”[MeSH Terms]) AND (“Mastication”[MeSH Terms] OR “chew*”[Title/Abstract] OR “masticat*”[Title/Abstract] OR “speech”[Title/Abstract] OR “swallow*”[Title/Abstract] OR “deglutition”[MeSH Terms] OR “function*”[Title/Abstract] OR “eating”[Title/Abstract] OR “comfort”[Title/Abstract] OR “salivation”[MeSH Terms] OR “Xerostomia”[MeSH Terms] OR “Mucositis”[MeSH Terms] OR “Stomatitis”[MeSH Terms] OR “Candidiasis, Oral”[MeSH Terms] OR “Xerostomia”[Title/Abstract] OR “Mucositis”[Title/Abstract] OR “Stomatitis”[Title/Abstract] OR “Candidiasis”[Title/Abstract] OR “Hyposalivation”[Title/Abstract] OR “Dry mouth”[Title/Abstract] OR “salivary flow rate”[Title/Abstract] OR “food oral processing”[Title/Abstract] OR “comminution”[Title/Abstract] OR “maximum bite force”[Title/Abstract] OR “maximum voluntary bite force”[Title/Abstract] OR “jaw muscle activity”[Title/Abstract] OR “masseter muscle thickness”[Title/Abstract] OR “tongue pressure”[Title/Abstract] OR “tongue force”[Title/Abstract] OR “lip force”[Title/Abstract] OR “mandibular movement”[Title/Abstract] OR “Oral stereognosis”[Title/Abstract] OR “oral diadochokinesis”[Title/Abstract] OR “oral tactile sensitivity”[Title/Abstract] OR “jaw kinematics”[Title/Abstract] OR “Denture use”[Title/Abstract] OR “Denture retention”[Title/Abstract] OR “Denture satisfaction”[Title/Abstract] OR “Quality of Life”[MeSH Terms] OR “oral health‐related quality of life”[Title/Abstract] OR “OHRQoL”[Title/Abstract] OR “Oral Health Impact Profile”[Title/Abstract] OR “OHIP”[Title/Abstract] OR “Oral Impact on Daily Performances”[Title/Abstract] OR “OIDP”[Title/Abstract] OR “Self‐reported oral health”[Title/Abstract] OR “Perceived oral health”[Title/Abstract])
	Scopus	(elder* OR “geriatric population*” OR “geriatric patient*” OR “aging population” OR “aged population” OR “aged 60” OR “60 year*” OR gerodont* OR “aged 75” OR “75 year*” OR “aged 80” OR “80 year*” OR senior* OR care‐depend* OR “older adults” OR “older people” OR “dental care for aged”) AND (“tooth loss” OR edent* OR “missing teeth” OR “jaw, edentulous”) AND (chew* OR masticat* OR speech OR swallow* OR deglutition OR function* OR eating OR comfort OR salivation OR xerostomia OR mucositis OR stomatitis OR candidiasis OR hyposalivation OR “dry mouth” OR “salivary flow rate” OR “food oral processing” OR comminution OR “maximum bite force” OR “maximum voluntary bite force” OR “jaw muscle activity” OR “masseter muscle thickness” OR “tongue pressure” OR “tongue force” OR “lip force” OR “mandibular movement” OR “oral stereognosis” OR “oral diadochokinesis” OR “oral tactile sensitivity” OR “jaw kinematics” OR “denture use” OR “denture retention” OR “denture satisfaction” OR “quality of life” OR “oral health‐related quality of life” OR ohrqol OR “oral health impact profile” OR ohip OR “oral impact on daily performances” OR oidp OR “self‐reported oral health” OR “perceived oral health”)
	Web of Science	(elder* OR “geriatric population*” OR “geriatric patient*” OR “aging population” OR “aged population” OR “aged 60” OR “60 year*” OR gerodont* OR “aged 75” OR “75 year*” OR “aged 80” OR “80 year*” OR senior* OR care‐depend* OR “older adults” OR “older people” OR “dental care for aged”) AND (“tooth loss” OR edent* OR “missing teeth” OR “jaw, edentulous”) AND (chew* OR masticat* OR speech OR swallow* OR deglutition OR function* OR eating OR comfort OR salivation OR xerostomia OR mucositis OR stomatitis OR candidiasis OR hyposalivation OR “dry mouth” OR “salivary flow rate” OR “food oral processing” OR comminution OR “maximum bite force” OR “maximum voluntary bite force” OR “jaw muscle activity” OR “masseter muscle thickness” OR “tongue pressure” OR “tongue force” OR “lip force” OR “mandibular movement” OR “oral stereognosis” OR “oral diadochokinesis” OR “oral tactile sensitivity” OR “jaw kinematics” OR “denture use” OR “denture retention” OR “denture satisfaction” OR “quality of life” OR “oral health‐related quality of life” OR ohrqol OR “oral health impact profile” OR ohip OR “oral impact on daily performances” OR oidp OR “self‐reported oral health” OR “perceived oral health”)
	Cochrane	(elder* OR “geriatric population*” OR “geriatric patient*” OR “aging population” OR “aged population” OR “aged 60” OR “60 year*” OR gerodont* OR “aged 75” OR “75 year*” OR “aged 80” OR “80 year*” OR senior* OR care‐depend* OR “older adults” OR “older people” OR “dental care for aged”) AND (“tooth loss” OR edent* OR “missing teeth” OR “jaw, edentulous”) AND (chew* OR masticat* OR speech OR swallow* OR deglutition OR function* OR eating OR comfort OR salivation OR xerostomia OR mucositis OR stomatitis OR candidiasis OR hyposalivation OR “dry mouth” OR “salivary flow rate” OR “food oral processing” OR comminution OR “maximum bite force” OR “maximum voluntary bite force” OR “jaw muscle activity” OR “masseter muscle thickness” OR “tongue pressure” OR “tongue force” OR “lip force” OR “mandibular movement” OR “oral stereognosis” OR “oral diadochokinesis” OR “oral tactile sensitivity” OR “jaw kinematics” OR “denture use” OR “denture retention” OR “denture satisfaction” OR “quality of life” OR “oral health‐related quality of life” OR ohrqol OR “oral health impact profile” OR ohip OR “oral impact on daily performances” OR oidp OR “self‐reported oral health” OR “perceived oral health”)
